# Layer-by-layer decoding of contemporary and historic painting composition using MALDI mass spectrometry imaging and machine learning

**DOI:** 10.1126/sciadv.adz4427

**Published:** 2026-03-25

**Authors:** Václav Krupička, Florent Grélard, Landry Blanc, Aleksandra Popowich, José Luis Lazarte Luna, Nicolas Desbenoit, Julie Arslanoglu, Caroline Tokarski

**Affiliations:** ^1^ Univ. Bordeaux, CNRS, Bordeaux INP, CBMN, UMR 5248, F-33600 Pessac, France.; ^2^ Univ. Bordeaux, CNRS, Bordeaux Proteome, UAR 2046, F-33076 Bordeaux, France.; ^3^ Department of Scientific Research, The Metropolitan Museum of Art, New York, NY, USA.; ^4^ Department of Painting Conservation, The Metropolitan Museum of Art, New York, NY, USA.

## Abstract

Layer-by-layer characterization of modern and historic paintings is essential for understanding how an artwork was created and how it has changed over time. This information can reveal historical or societal insights, inform attribution and classification, and support preservation efforts. The determination of the identity, structure, and composition of art material remains challenging due to the complex, multilayered nature of paintings. These structures often contain mixtures of organic and inorganic components, with unknown in situ chemical interactions, that create cross-linked and chemically modified molecular networks and assemblies. In this work, we have developed a method with high chemical specificity to identify and map organic and inorganic components in modern and historic multilayered paint systems. Our approach achieves an unprecedented level of molecular detail using a single technique. In addition, we have designed the method for automated composition assignment of individual layer through high-resolution matrix-assisted laser desorption/ionization (MALDI) mass spectrometry imaging (MSI) and a dedicated composition prediction model.

## Teaser

MALDI-MSI with machine learning identifies and maps organic and inorganic compounds in historic painting cross sections.

## INTRODUCTION

Identifying and mapping the molecular components of cultural heritage (CH) objects is vital for advancing our understanding of artworks. By creating a spatial image of the composition and complex chemistries occurring within the layers of artwork, we gather information not only on artist materials, but also on conservation history, state of preservation, and attribution. In addition, we can often situate objects within a specific time, space, and historical context based on its materiality, revealing societal, cultural, and economic insights of past artists or makers. Artworks and museum objects are more than assemblages of materials: They are complex, often multilayered compositions of mineral pigments, organic colorants, and binders such as oils, waxes, gums, proteins, or synthetic polymers. These materials are purposefully selected by artists based on tradition, experience, and intended visual effects (see the Supplementary Materials on the structure of paint cross sections). These materials are not static: They undergo chemical modifications to achieve specific working, mechanical, or optical properties and continue to interact with each other and their environment over time, creating intricate cross-linked and chemically modified molecular networks of binders and pigments.

To date, chemical analysis in heritage science has primarily focused on identifying inorganic and organic constituents. While substantial advancements have been made in inorganic material studies (*1*), the analysis of organic materials has often been limited to the detection of the organic molecule type or limited structural identification. The ability to investigate organic macromolecules has more recently emerged with advances in analytical chemistry and mass spectrometry (*2*–*4*). The need to explore the diversity and complexity of organic macromolecules, using state-of-the-art analytical techniques and multimodal analysis for organic and inorganic compounds, integrated within an art historical and conservation framework, is becoming increasingly evident in CH studies.

Current strategies for mapping organic and inorganic molecules rely primarily on spectroscopic techniques (*2*, *5*–*7*), which identify elemental and chemical bond compositions, but often lack chemical specificity. To date, the most widely used mass spectrometry imaging (MSI) technique in CH studies is time-of-flight secondary ion mass spectrometry (TOF-SIMS). While TOF-SIMS effectively identifies and maps organic and inorganic molecules (*2*, *8*–*11*), its high-energy ionization process often fragments large fragile organic molecules, such as proteins, significantly limiting the information that can be extracted (*12*).

In this work, we present the first application of a matrix-assisted laser desorption/ionization (MALDI) MSI workflow for studying historic paintings. MALDI-MSI enables untargeted, label-free analysis of a wide range of molecular species, from small molecules such as lipids, to large macromolecules such as proteins (*13*–*16*), with high spatial resolution—down to 5 μm in this study—and unparalleled chemical specificity (*17*). MALDI-MSI is particularly relevant for analyzing multilayered and multicomponent materials such as paintings. Early attempts have explored painting replicas containing reference materials targeting the study of low–molecular weight molecules such as pigments (*18*, *19*). Here, we demonstrate how our MALDI-MSI method can decode the molecular organization of paint layers by identifying and mapping both high– and low–molecular weight ions for structure characterization. This technique allows us to locate and monitor intact materials, by-products resulting from in situ degradation, and inorganic/organic complexes within samples—at the surface, within layers, and at layer interfaces—offering insights into artwork composition and preservation.

## RESULTS

### Developing the MALDI-MSI workflow for paints and building a custom pigment and dye database for spectrum annotations

While MALDI-MSI was originally developed for biological applications, adapting it for CH materials requires significant modifications. Each step in our workflow for analyzing paint samples has been carefully optimized, from the sectioning method used to prepare thin cross sections of samples from artworks, to the MALDI-MSI settings that enable simultaneous screening of heterogeneous molecular classes. In addition, we have developed a custom machine learning assignment model, MSIpredictART, to enhance molecular identification.

**Fig. 1.**
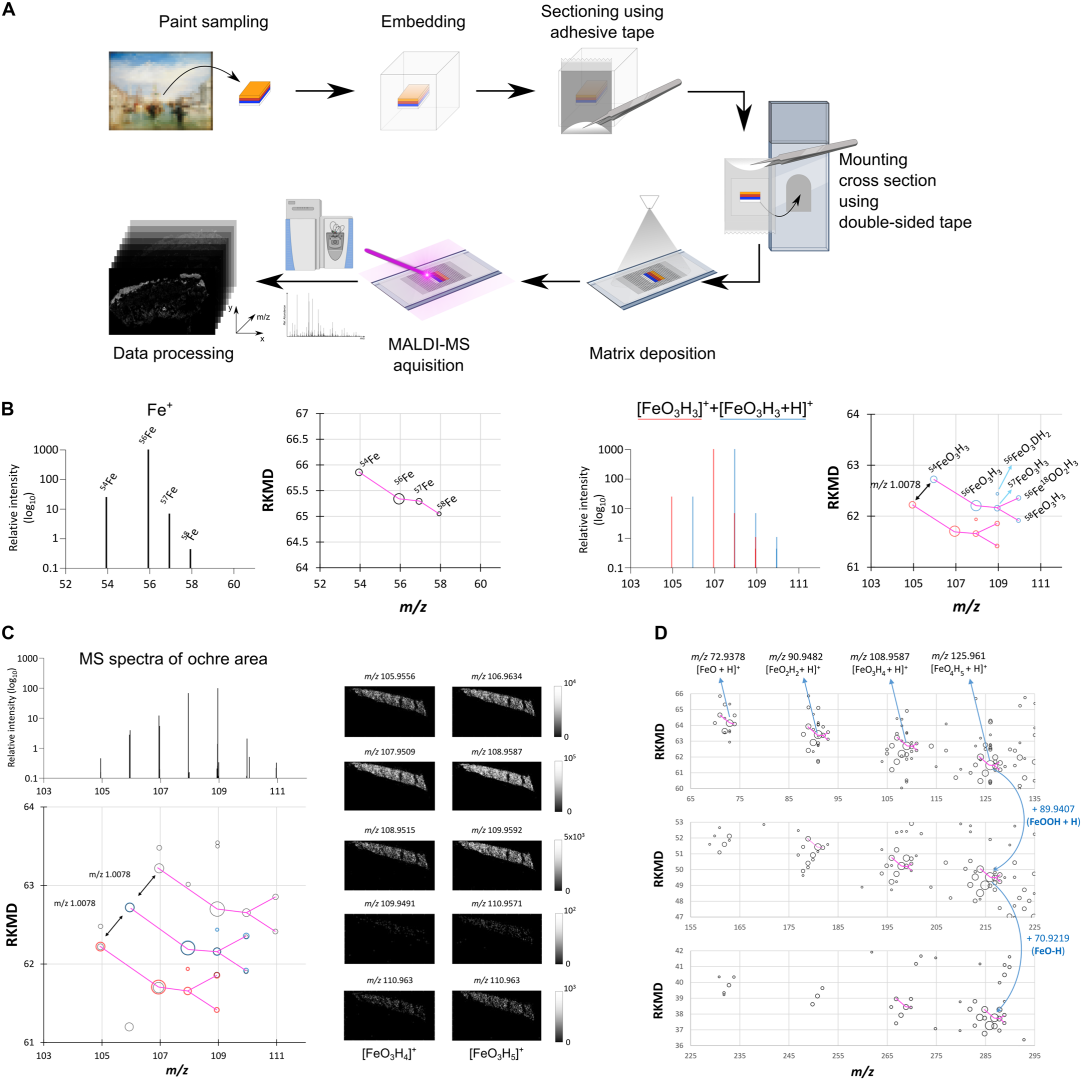
Acquisition workflow and KMD visualization. (**A**) Optimized workflow for preparing painting cross sections using commercially available adhesive tape for acquiring MSI. (**B**) Theoretical isotopic profiles of iron and iron-containing species, illustrating their unique “fork-like” 2D patterns in referenced KMD (RKMD) plots. (**C**) Observed iron 2D pattern in the yellow ochre pigment region of Pratt2 C4 dataset (see LASSO prediction images in fig. S3). (**D**) Pigment-related peaks identified in RKMD for yellow ochre, highlighting successive additions of FeOOH and water losses.

Currently, no standardized protocol exists for preparing paint cross sections compatible with MALDI-MSI analysis. Several challenges arise when adapting traditional sectioning protocols to artwork samples. Painting samples are highly heterogeneous and brittle, often curling or disintegrating during sectioning. Furthermore, given the irreplaceable nature of artworks, destructive sampling must be minimized to prioritize the preservation. The available sample sizes are exceptionally small, with paint layers often measuring a few micrometers in thickness. To address these challenges, we developed an optimized workflow (Fig. 1A) that uses common stationary adhesive tape. This tape serves a dual purpose: First, it stabilizes the paint cross section, preventing crumbling or distortion during sectioning; second, it securely attaches the cross section to a glass slide using double-sided tape, enabling matrix deposition and MALDI-MSI analysis.

Our sample training set consisted of 15-year-old multilayer paint replicas prepared using traditional recipes, with well-documented stratigraphies (table S1). Notably, each pigment and binder used is not a single chemical entity; table S2 details the chemical compositions of the materials studied. Since no dedicated database exists for annotating MSI data related to art materials, we have so far relied on databases originally developed for biomedical applications. To address this limitation, we created a custom pigment and dye database for this study using PubChem entries and uploaded it to the free web application METASPACE2020 (*20*). This database allows for the assignment of a wide range of pigment compositions in paint replicas. For example, analysis of the Pratt2 A4 dataset, the database search identified ferric oxide [mass/charge ratio (*m*/*z*) 106.943] and lead oxide (*m*/*z* 262.935), confirming the presence of ochre and lead white paint layers, respectively, in agreement with the known composition of Pratt2 A4 paint mockup (see P2A4 composition in table S1).

### Kendrick mass defect plot to visualize pigment molecular distributions

Inorganic pigment components in our datasets can be further studied beyond database-based identification through their unique isotopic distributions using Kendrick mass defect (KMD) plots (*21*). KMD analysis is a mass spectrometry approach in which ion masses are rescaled relative to a chosen reference unit, allowing chemically related or isotopically patterned species to be visualized together. KMD plots reveal characteristic isotopic two-dimensional (2D) patterns for pigments based on theoretical isotopic profiles. For example, iron-containing pigments exhibit a distinctive “fork-like” pattern (Fig. 1B). This unique isotopic signature can be tracked across the *m*/*z* range, enabling the identification of additional pigment-related signals and providing a straightforward way to distinguish pigment-derived ions from other signals. We observed this pattern in several earth pigment–containing replicas, including the Pratt2 C4 sample (Fig. 1C), where we confirmed the presence of yellow ochre pigment (FeOOH·H_2_O) in layer 3 (see layer composition in table S1). Additional groups of pigment-related peaks were identified in Pratt2 C4 (Fig. 1D), displaying mass shifts corresponding to successive water losses and/or pigment cluster extensions by additions of FeOOH. The assigned molecular structures were validated by tandem mass spectrometry (MS/MS) analysis confirming *m*/*z* 125.961 identified as [FeO_4_H_5_ + H]^+^ (see MS/MS spectrum in fig. S1).

### Building the database for macromolecule analysis

Determining binder compositions is a substantial challenge, as it requires identifying macromolecular compounds such as proteins or individual components in highly cross-linked networks. These molecular species often undergo chemical modifications during the formulation and drying of paints and degrade over time (*3*, *4*), increasing the likelihood of misidentifications during database matching. To enhance molecular identification, we supplemented our custom database with SwissLipids (*22*) and the Human Metabolome Database (*23*) (HMDB 4.0) via METASPACE2020 to identify biomolecules in historic paint binders. While these databases provided valuable initial insights into paint layer compositions, they did not allow us to unambiguously differentiate between different binder types.

### Building the predictive model: MSIpredictART

**Fig. 2.**
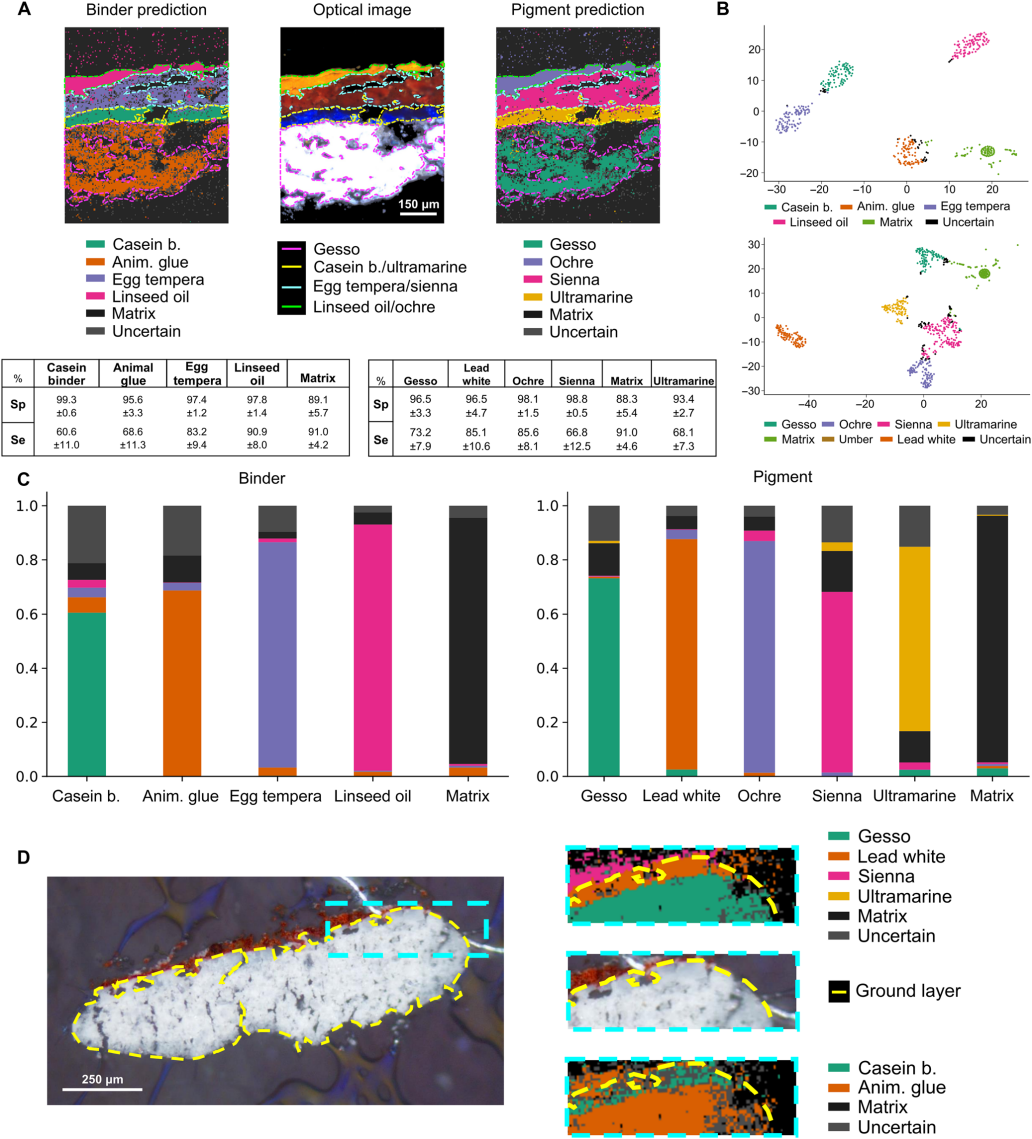
LASSO model for paint layer composition identification. (**A**) Brightfield (BF) image showing the highlighted region of interest with known composition (center) alongside LASSO/GMM model predictions for binder (left) and pigments (right). Specificity (Sp) and sensitivity (Se) values for each class across all datasets are shown below. (**B**) 2D *t*-SNE visualization of high-dimensional binder/pigment data from training and validation datasets, with pixels removed by GMM clustering highlighted in black. (**C**) Bar chart illustrating the prediction matrix for binder (left) and pigment (right). (**D**) BF image of Pratt3 D4 training dataset (left) with an inset showing corresponding LASSO model predictions for binder and pigment, successfully identifying an optically indistinguishable casein/lead white layer. (A to C) is related to Pratt2 D3 and (D) to Pratt3D4. Anim. glue, animal glue; casein b., casein binder.

To differentiate between organic binder classes, in particular, protein-based binders, we have developed a supervised machine learning algorithm to efficiently and automatically annotate both binder and pigment layer compositions, streamlining CH MALDI-MSI data analysis. Our predictive model, MSIpredictART, is built using the least absolute shrinkage and selection operator (LASSO), which has previously been successfully coupled with MSI (*24*). Compared with other regression techniques, LASSO yields sparse models that rely only on the most relevant ion features, making the resulting models simpler and easier to interpret. Mathematical weights are assigned to ion features, highlighting peaks whose presence or absence is most informative for distinguishing classes. The selected ion features may serve as potential biomarkers for specific binders or pigments or reflect protein degradation pathways; however, a systematic identification and structural assignment of these ions was beyond the scope of this study and is currently being investigated in our group. The LASSO-based model enables automatic identification and prediction of binder/pigment compositions within paint layers without relying on individual ion signatures (Fig. 2A). One potential challenge in analyzing historical paint samples is that artist materials vary greatly and can include nontraditional, local, or historic sources. To account for unknown binders and pigment compositions not included in the training sets, we introduced a supplementary “Uncertain” class using Gaussian mixture model (GMM). LASSO prediction scores are clustered into classes using the GMM model, and only pixels with ≥95% probability of belonging to a class are retained; lower-confidence pixels are filtered out and placed into the Uncertain category. Results after GMM clustering can be visualized using *t*-distributed stochastic neighbor embedding (*t*-SNE) in Fig. 2B, where predictions classified as Uncertain are highlighted in black. In addition, we incorporated a “Matrix” class that represents pixels from sample sets that do not fall into other masks, ensuring that off-sample signals are appropriately categorized.

To evaluate the quality of these assignments, we compared the predicted binder/pigment classes to annotated regions extracted from aligned optical images. Specificity (true negative rate) and sensitivity (true positive rate) were calculated based on the agreement between predicted pixel classes and the ground-truth optical masks, with detailed values for each dataset provided in the Supplementary Materials (tables S3 and S4).

Our LASSO model demonstrates high specificity and sensitivity (Fig. 2, A and C) with specificity exceeding 90% for pigments and 95% for binders, providing strong assignment confidence. Sensitivity levels ranged from 60 to 90% (fig. S2 and tables S3 and S4). The model can also differentiate regions in the paints where defects, such as voids, are present. The inclusion of Matrix and “Unknown” classes significantly reduces the potential for false assignments. In Fig. 2C, we present the distribution of predictions for each binder and pigment class included in the LASSO model. Most pixels are correctly assigned, with a portion designated as Matrix or Uncertain (represented in black and gray, respectively). Notably, nearly all pixels in the Matrix class were accurately predicted, highlighting the minimal occurrence of off-sample artifacts. This can be further illustrated using the ochre and lead white layers in the Pratt2 A4 sample, where MSIpredictART successfully assigned the correct classifications (see violet and orange layers in P2A4 pigment image, fig. S3).

### Layers’ composition correctly assigned using MALDI-MSI workflow and MSIpredictART machine learning tool

The predictive model’s performance was assessed using a set of replicas from our test dataset. These samples were chosen specifically because they contained optically indistinguishable layers (they are the same color), which prevented the extraction of optical masks necessary for training.

We highlight this with the Pratt3 D4 dataset, which consists of three distinct layers, two of which cannot be visually distinguished. Using MSIPredictArt, each layer’s composition was correctly assigned (Fig. 2D), including the differentiation of the two white paint layers. The model identified the upper layer as containing casein binder and lead white pigment, while the underlying layer was correctly assigned as containing animal glue and gesso (see table S2 for composition of pigments and binders). This demonstrates our model’s capability to accurately reveal and identify layer compositions, even in cases where visual inspection/microscopy alone is insufficient. MSIpredictART predictions for all paint cross sections are provided in fig. S3.

### MALDI MSI workflow maps varnish penetration in the layers

A subsection of our paint models contained an additional varnish layer applied over the final paint layer. Varnishes serve as protective coatings and enhance color effects in paintings. However, yellowing of natural resin or oil varnishes over time can alter the appearance of a painting and necessitate consideration of their removal. Understanding the composition and location of a varnish layer is important for selecting appropriate conservation approaches to avoid unnecessary damage to underlying paint layers.

In this study, the varnish used was dammar resin dissolved in turpentine. Dammar resin is a triterpenoid resin consisting of a complex mixture of cyclic isoprenoid compounds and a polymeric fraction, based on polycadinene (*25*). The varnish layer was not directly observable with a microscope; i.e., very thin and optically transparent. However, using MALDI-MSI, we identified terpenoids and terpenoid derivatives associated with dammar resin on the outer edge of the topmost paint layer. Specifically, we detected sodium adducts of ursolic (or oleanolic) acid, dammarenolic acid, hydroxyoleanonic lactone, schoreic acid, and asiatic acid, all previously reported as markers in studies of aged dammar varnishes (*26*). In addition, we observed that these marker compounds had penetrated the topmost paint layer within their respective datasets, highlighted in fig. S4. This raises important questions regarding the feasibility of complete varnish removal during conservation treatment and whether the uppermost paint layer is permanently compromised if impregnated with aging varnish.

### Layer-by-layer identification and mapping of organic and inorganic high– and low–molecular mass compounds in historic artwork

We applied our MALDI-MSI workflow and trained predictive model to a historic sample from *The Marriage of the Virgin* by José Sanchez (The Metropolitan Museum of Art, accession no. 2016.553, approximately 1690). A description of the artwork is provided in the Supplementary Materials. This represents the first known application of MALDI-MSI analysis on a historic artwork and is among the first examples of simultaneous organic and inorganic mapping and identification of artist materials in a single measurement. As such, it marks a substantial step forward in the characterization of materials in paints and artworks of unknown composition, including materials not incorporated in our training set and those altered over time. To validate and support our MALDI-MSI findings, we conducted a comprehensive set of additional analyses, to confirm the artist materials, using scanning electron microscopy–energy-dispersive x-ray spectroscopy (SEM-EDS), Raman spectroscopy (*27*, *28*), attenuated total reflectance–Fourier transform infrared spectroscopy (ATR-FTIR), pyrolysis–gas chromatography–mass spectrometry (Py-GC-MS), and liquid chromatography tandem mass spectrometry (LC-MS/MS). A summary of the combined data obtained from MALDI-MSI and these complementary techniques is provided in the Supplementary Materials and table S5. Unless otherwise noted, all MALDI-MSI results corroborate with these complimentary analyses demonstrating the power of MSI to provide the layer-by-layer structure and composition of both the inorganic and organic artist materials in a historic painting.

**Fig. 3.**
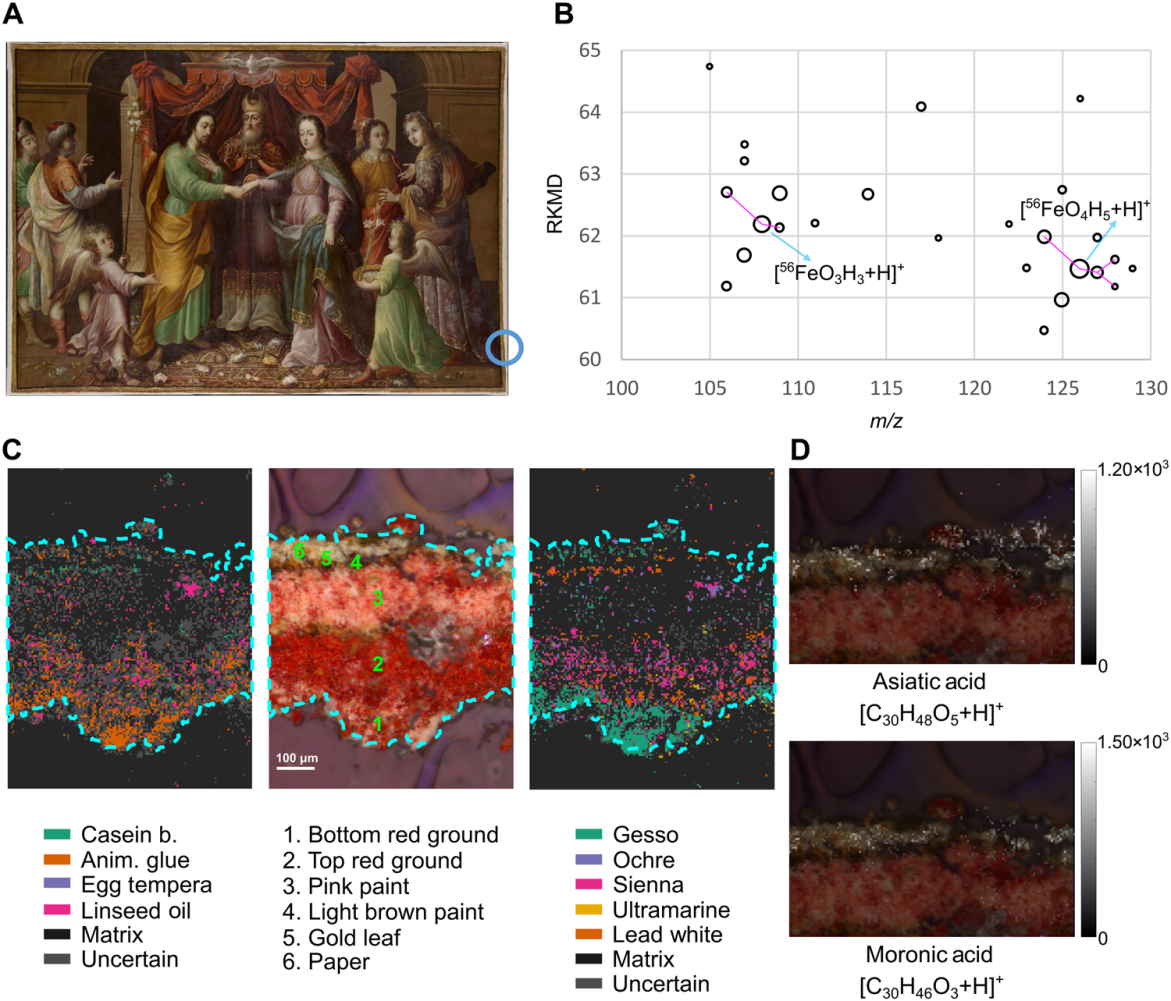
MALDI-MSI analysis of *The Marriage of the Virgin* by José Sanchez (approximately 1690, The Metropolitan Museum of Art, New York, accession number 2016.553). (**A**) Sampling location within the painting. (**B**) RKMD plot of the extracted spectra for second red ground area (no. 2). (**C**) BF microscopy image with corresponding LASSO predictions for binder and pigment. Optically distinguishable layers in the microscopy image are numbered, with their compositions detailed in table S1. (**D**) Ions associated with the presence of mastic or dammar varnish that permeated into the paper layer.

Both KMD analysis (Fig. 3B) and LASSO prediction (Fig. 3C), as described previously, were applied to determine the composition of painting. The sample was taken from the painting’s proper left edge (Fig. 3A). Microscopic examination of the paint cross section revealed two ground layers (layers 1 to 2), multiple paint layers (layers 3 to 4), gilding (layer 5), and evidence of prior conservation treatment (paper strips applied during canvas relining, layer 6). Layer 1 was assigned as gesso by MSIpredictART, this correlates with previous SEM-EDS analyses (*27*, *28*), which identified a unique calcium-rich ground material composed of calcite (calcium carbonate) pseudomorphs, the byproduct of potassium extraction from wood ash to produce lye, and iron earth pigments (*28*). Given that the paint replicas used to train the predictive model contained a gesso mixture of calcium carbonate and calcium sulfate (confirmed by SEM-EDS), the gesso assignment for the calcium carbonate layer is valid. The binder for layer 1 was identified as animal glue, correlating with the identification of collagens, which are the major component of animal glue, identified by bulk (nonspatial) LC-MS/MS.

In layer 2, MSpredictART indicated the presence of an animal glue and an oil binder. As seen by SEM-EDS, MSIpredictART (Fig. 3C) assigned an iron oxide-containing pigment, sienna, to layer 2, with lead white. In addition to the predictive model, KMD analysis was applied (Fig. 3B), revealing patterns consistent with specific iron oxide species in layer 2, corroborating previous findings in paint replicas including the identification of FeO(OH)·2H_2_O at *m*/*z* 125.961 (fig. S1), providing information on the molecular structure not gained by SEM-EDS or the predictive model.

Layer 3, likely an organic red lake not included in the training set for the model mixed with calcium sulfate, was not assigned by MSpredictART, possibly due to low signal intensity in this layer and low overall signal-to-noise. Pixels in this layer were instead classified as Matrix and Unknown rather than falsely assigned to an incorrect material, demonstrating the model’s robustness in handling unfamiliar materials or poor-quality data. This Unknown result underscores the value of expanding the training dataset to include a broader range of historical pigments. While our current model successfully covers all major binder types and key pigments, the inclusion of additional pigments will further enhance the model’s accuracy and applicability across diverse artworks.

Layer 4 identified as lead white by the predictive model, supporting its identification as a pigment in the light (brown in cross section) paint. MALDI-MSI data for layer 5 revealed the presence of ultrathin gold leaf, detected at *m*/*z* 196.967 (fig. S5); although this layer is <1-μm thick (measured by SEM), it can still be identified by MSI using a 5-μm pixel size. Elsewhere, SEM-EDS analyses also showed the presence of an uneven calcium layer colocalized with the paper conservation material (layer 6), which was assigned as gesso by MSIpredictART. Last, layer 6 also contains an additional artist material, a mastic or dammar varnish, not included in the model, which can be seen in the extracted ion maps for the varnish markers of asiatic acid (*m*/*z* 511.339) and moronic acid (*m*/*z* 455.353) shown in Fig. 3D. The varnish was applied to *The Marriage of the Virgin* following relining and the addition of the paper strips along the tacking edges, permeating through the paper (layer 6).

### Layer-by-layer identification and mapping of organic and inorganic high– and low–molecular compounds in contemporary artwork

**Fig. 4.**
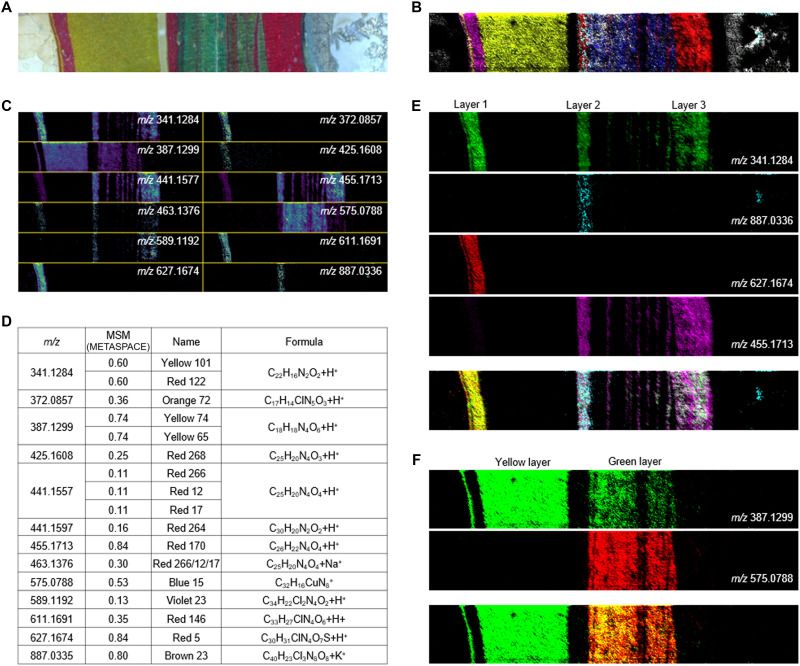
Pigments composition identified using pigment database. (**A**) Optical image of the studied paint cross section. (**B**) False color image of based on identified pigment ions, demonstrating that a subset of ions observed in MSI data can be used to interpret painting structure. (**C**) Ions annotated using a custom-built pigment database implemented on METASPACE2020. (**D**) Corresponding pigment names and structures for the annotated ions. (**E**) Images of color bands identified through MSI for PR122 (341.1284 *m*/*z*), PBr23 (887.0336 *m*/*z*), PR5 (627.1674 *m*/*z*), and PR170 (455.1713 *m*/*z*). (**F**) The green band resulting from the mixing of PY74/65 (387.1299 *m*/*z*) with PB15 (575.0788 *m*/*z*) pigments.

The second artistic sample studied was a cross section from a contemporary painting by a local Bordeaux artist, shown in Fig. 4A. Our analysis revealed a complex pigment composition, with 17 distinct pigment species present, including isobars (listed in Fig. 4D). In particular, the three red layers, highlighted in Fig. 4E, exhibited highly nuanced pigment compositions. Red layer 1 contained a mixture of pigment red 5 (PR5) and PR122, while red layer 2 consisted of a mixture of PR122, PR170, and pigment brown 23 (PBr23). Red layer 3 was composed of a blend of PR122 and PR170, delineating the stratified complexity of red hues as the artist intentionally blended paints to achieve the desired color effect. Similarly, the ostensibly green layer in Fig. 4F [as observed in the brightfield (BF) microscopy image] was not created using a green pigment but rather through the mixing of pigment yellow 74/64 (PY74/64) with pigment blue 15 (PB15). These results highlight the ability of MALDI-MSI to provide detailed insights into pigment/paint mixing within layered paint systems due to its high chemical specificity.

**Fig. 5.**
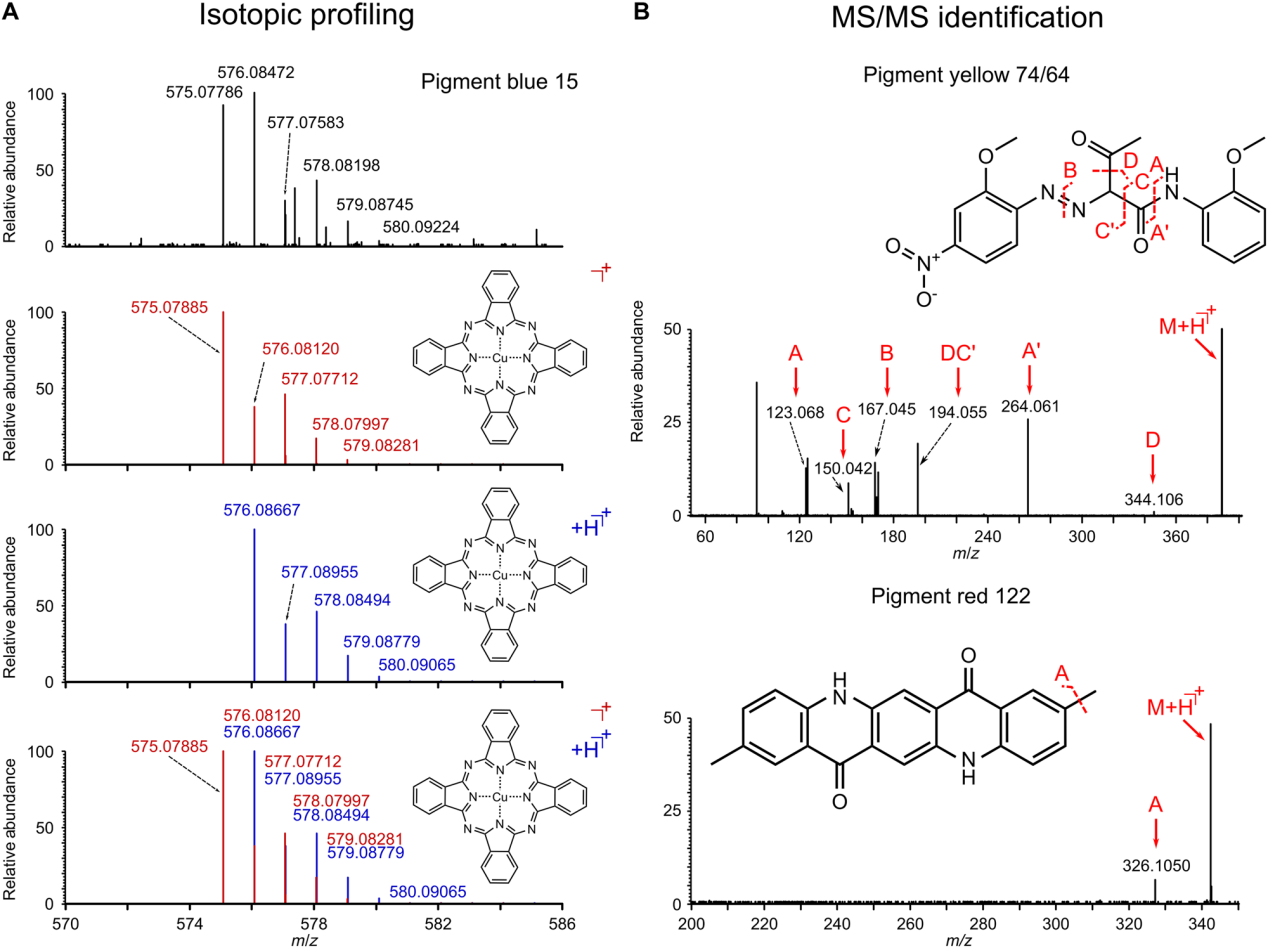
Structural analysis and identification of pigments. (**A**) Isotopic profiling enables the verification of pigment species by leveraging the unique isotopic distribution of metallic compounds. (**B**) MS/MS analysis using higher-energy collisional dissociation (HCD) fragmentation provides additional structural information for verification. In isotopic profiling, experimental spectra (top in black) are compared to theoretical isotopic distributions for M^+^ and [M + H]^+^ species. MS/MS analysis further enhances structural information by revealing fragmentation patterns, such as for PY74/64 and distinguishing between isobaric species, as demonstrated for PR122 and PY101.

Confidently identifying pigment compositions in the presence of isobaric species, as seen in this sample, necessitates additional analyses. Isotopic profiling is particularly useful for elements with highly specific isotopic distributions, such as those containing Cu, Fe, or Pb, demonstrated here in the case of PB15 in Fig. 5A. Theoretical isotopic profiles (red for M^+^ and blue for [M + H]^+^) are compared to experimental spectra, providing a strong basis for molecular identification. Alternatively, MS/MS experiments allow a more detailed molecular structural analysis, as exemplified in the case of PY74/64. MS/MS also helps distinguish two isobaric pigment species as in the case of PY101 and PR122 (Fig. 5B). The ability of MALDI-MSI to provide precise mass assignments while offering deeper structural insights sets it apart from all currently used mapping techniques in CH.

## DISCUSSION

By combining MALDI-MSI with machine learning, we can identify and map a wide range of organic and inorganic artist materials in paintings, polychromy, and textiles, spanning from historic to contemporary periods. Here, we present the first study of a historic painting by MALDI-MSI. Our workflow provides layer-by-layer characterization of binder and pigment compositions from a single analysis. The results we obtained are comparable to data from a series of spectroscopic (SEM-EDS, Raman, and ATR-FTIR) and bulk mass spectrometry (Py-GC-MS and LC-MS/MS) experiments (see table S5) and preserve valuable spatial information. Furthermore, the workflow can expand to include additional data analysis approaches, some of which include (i) KMD to provide information on molecular species, as shown for the red iron earth pigment FeO(OH)·2H_2_O in *The Marriage of the Virgin* (Fig. 3B); (ii) extracting ion maps for specific isotopes, here allowing for identification of gilding layers (fig. S5); (iii) secondary database searches such as our custom-built pigment database applied to the contemporary painting (Fig. 4); and (iv) additional structural analysis using isotopic profiling and MS/MS for pigment confirmation (Fig. 5). All of these MSI approaches can be applied to a single sample while maintaining stratigraphic information, ensuring minimal sampling of irreplaceable CH objects while garnering maximum information.

Of note, distinguishing between different organic binders, such as oils, proteins, and resins, is a common question when studying or conserving paintings or polychrome objects. MALDI-MSI provides the ability to directly differentiate these binders in situ, providing critical insights into artistic techniques, material selection, and workshop practices, in addition to identifying conservation treatments, which in turn aids authentication, dating, and attribution of CH objects. Furthermore, our predictive model, MSIpredictART, excels at differentiating binders of proteinaceous origin (casein, tempera, and animal glue), a common question when studying and conserving paintings and polychrome objects and an analytical limitation of other spatially resolved techniques (ATR-FTIR and Raman).

Beyond binder identification, this platform represents a major advancement in the analysis of historical and contemporary paintings. MALDI-MSI of *The Marriage of the Virgin* by José Sanchez marks the first analysis of a historic painting, demonstrating the power of this technique to identify organic binders, pigments, varnishes, and even submicrometer gilding (Fig. 3 and fig. S5) with high spatial resolution on a sample of size comparable to traditional cross sections taken from an artwork. These results provide a comprehensive picture of how the artist built up the layers of the painting: Sanchez’s technique is in line with those found in paintings by other Mexican artists from the late seventeenth century using a double ground containing animal glue (mixed with oil in the second), red iron oxides, and calcite (a by-product of lye production from plant ash), a tradition that central Mexico shared with Madrid (*26*, *27*), as well as several paint layers and a gilded trompe-l’œil frame. In addition, MALDI-MSI (Fig. 3D) was able to identify later conservation treatments, demonstrating that revarnishing had caused varnish to penetrate through another layer (paper stripes, layer 6). Similarly, an untitled work by a contemporary Bordeaux artist showed the broad applicability of MALDI-MSI to identify modern pigments (Fig. 4) in highly layered cross sections, with the addition of MS/MS structural confirmation (Fig. 5). This revealed the artist’s preference for varying paint layer thickness, likely painting wet on wet in the thinnest layers (Fig. 4F, green layer), and either the manufacturer’s or artist’s preference for mixing pigments to expand their palette. As demonstrated, in addition to its applications in historical artwork analysis, the MALDI-MSI platform holds notable potential for the study and preservation of contemporary art. Use of unconventional materials, synthetic binders, mixed media, and complex layering techniques by modern and contemporary artists often poses a substantial challenge when using traditional analytical methods. The ability of MALDI-MSI to spatially resolve and chemically identify a wide range of organic and inorganic components makes it particularly well suited for characterizing these often nontraditional materials. This is especially valuable in documenting the original material state of contemporary works, many of which are prone to rapid aging or material instability. By providing detailed molecular maps of these artworks, our platform can guide preventative conservation and provide insight into molecular changes occurring during ageing of these complex media.

The evolutive nature of our predictive model allows for continuous improvements, as additional datasets can be incorporated into training, enhancing the model’s robustness and expanding its predictive capabilities. Furthermore, to maximize the spatially resolved chemical information generated through this approach, MALDI-MSI should be integrated in a multimodal framework alongside other imaging methods (SEM-EDS, Raman, and ATR-FTIR) used in material characterization. As CH samples from paintings, polychromy, and textiles are already frequently studied in cross section to understand an artist’s layering technique, characterize the individual materials, and investigate the evolution of an object over its lifetime (degradation or addition of conservation treatments), the addition of MALDI-MSI into a multimodal framework would enable the creation of highly complementary molecular maps, enriching our understanding of paint systems and their compositions and improving our understanding and contextualization of artwork, without requiring larger/additional sampling. While in some cases, MALDI-MSI may completely negate the need for multiple techniques, it is currently limited to a spatial resolution of around 5 μm, which nonetheless allows detailed, layer-by-layer chemical mapping. MALDI-MSI is particularly suited to scientists studying complex materials composed of layered inorganic and organic components in a dried or cured organic matrix, which may be challenging to analyze by other methods. Likewise, it provides CH researchers with a powerful, user-friendly tool for mapping both organic and inorganic materials in paintings, offering deeper insights into artistic techniques and material choices and informing conservation strategies.

For museums and CH institutions, the capacity to generate high-resolution molecular maps enhances collection management by supporting provenance research and informing conservation strategies based on detailed material composition. In addition, the ability to differentiate original materials from later restorations promotes ethical conservation practices and helps maintain the historical integrity of artworks.

## MATERIALS AND METHODS

### Paint samples

A set of samples was obtained from multilayer paint replicas that were prepared at the Pratt Institute (Brooklyn, New York, USA) in 2008 using traditional artistic methods and modern pigments and binders. The wooden boards used as supports were first primed using animal glue followed by an application of a ground (preparation) layer composed of animal glue and gesso (mixture of CaCO_3_ and CaSO_4_). Various paint layers with protein binder were applied and left to dry (minimum of 10 days) before subsequent layers were applied on top. Linseed oil–containing layers were applied as the topmost layer 5 months after application of the last protein-based paint layer. The paint replicas contained common historic paint binders: animal glue, casein, whole egg tempera, and linseed oil. A range of historically common pigments was used in the replicas including sienna, ochre, lead white, and ultramarine (table S1). The studied historic paint sample was taken from *The Marriage of the Virgin* (accession no. 2016.553) by José Sanchez from The Metropolitan Museum of Art, New York, USA and chemically characterized by Met scientists (Supplementary Materials), allowing for method validation.

### Sample preparation

Samples from the replicas and historic painting were embedded in 7% carboxymethylcellulose (CMC) and sectioned using a CryoStar NX70 (Thermo Fisher Scientific, Germany). Common stationery tape (Maped Office, France) was attached by the adhesive side to the sample CMC block, completely covering the sample block face, and the sample was sectioned then in one slow, continuous motion. The 12-μm-thick sample cross section was attached to a glass slide, adhesive side up, using double-sided adhesive tape. BF microscopy images were acquired using a Leica DM2700M microscope (Leica Microsystems GmbH, Germany).

2,5-Dihydroxybenzoic acid (DHB) matrix was applied using a TM-Sprayer (HTX Technologies, North Carolina, USA) combined with an high-performance liquid chromatography pump (UltiMate, LC Packings, California, USA). A matrix solution containing DHB (20 mg/ml) in 70:30 (v/v) propanol/toluene, respectively, was sprayed with the following parameters: nitrogen flow (10 psi), 20 passes in a crisscross pattern with 3-mm offset, spray velocity (1200 mm/min), and isocratic flow of 50 μl/min. The amount of matrix deposited was calculated to be 4.8 μg/mm^2^.

### MALDI-MSI

MALDI-MSI was performed using an atmospheric pressure scanning microprobe MALDI (AP-SMALDI5, TransMIT GmbH, Giessen, Germany) source coupled to an Orbitrap Q-Exactive (Thermo Fisher Scientific GmbH, Bremen, Germany) mass spectrometer at a mass resolution power of 70,000 (full width at half maximum at *m*/*z* 400). Cross sections were analyzed in positive ionization mode over a mass range of 60 to 900 *m*/*z*. Acquisition was performed in 2D line mode with 3.7 pixels/s scan rate with 95-Hz laser frequency and 26 shots per pixel (38 nJ per shot) with stage step size of 5 μm. The laser attenuator was set at 35%. A source voltage of 4.5 kV was used with s-lens set to 90, and source capillary was heated to 350°C. Lock mass was used based on the theoretical signal of the DHB matrix (290.06591 *m*/*z*).

### Data analysis

MSI data were converted from proprietary Thermo Fisher Scientific “.raw” files to “.imzML” using RAW2IMZML converter v1.3R85 (TransMIT GmbH, Gießen, Germany). MSI datasets were preprocessed using Esmraldi; datasets were subjected to peak picking with prominence of 500 and 10 parts per million (ppm) step size to discard noisy signal. Local maxima were selected based on their relative height with respect to their neighbors (*29*). Spectral alignment with bin size of 10 ppm was carried out. MSI datasets were uploaded and annotated on METASPACE2020 using a custom pigment and dye database created from PubChem, SwissLipid, and the HMDB 4.0 entries.

### Data preprocessing for MSpredictART

MSpredictART was developed using training and validation datasets that consisted of 12 and seven paint cross sections from the Pratt models, respectively (table S1). Each cross section was unique, of known composition, and imaged using both MSI and BF optical microscopy. The images were preprocessed for use with the LASSO method using the following steps: MS images were normalized by the total ion count and scaled between 0 and 1. BF and MS images were co-registered manually, using a similarity transformation (scaling, rotation, and translation). The registration quality was assessed visually by comparing pixel-wise differences between images.

### Composition prediction using MSIpredictART

The LASSO approach was applied using MALDI-MS ion images as predictor variables (*X*) and regions from BF images as response variables (*Y*). After image registration, regions were extracted from BF images by color thresholding, where each region corresponds to a binder and a pigment. Regions were refined using linear regression to find the linear combination of ions that best approached the optical mask (fig. S6). Regions were sampled randomly, under the constraint of using the same number of pixels for each binder/pigment. The LASSO shrinkage parameter (μ) was determined by finding the minimum of the mean square error (MSE) between the theoretical and predicted images in the validation dataset. The shrinkage parameters used were 5 × 10^−5^, 1 × 10^−4^, 5 × 10^−4^, 1 × 10^−3^, 2 × 10^−3^, 3 × 10^−3^, 4 × 10^−3^, 5 × 10^−3^, 1 × 10^−2^, and 5 × 10^−2^. The minimum of the MSE was found at μ = 2 × 10^−3^. A new LASSO model was generated using this parameter, including both the training and validation datasets. The resulting model was obtained by bootstrapping and averaging over 10 repetitions. To identify and place low-confidence predictions into a separate class, GMMs were fit onto the predictions from the 12 training datasets. The final GMM was obtained by averaging those models. GMMs output probabilities of being predicted as each class of the model. An Uncertain class was defined for all pixels where the highest probability of belonging to any given class was <95%. The benefit of using GMM is evaluated by comparing the predictions from the GMM model and from thresholded LASSO. Uncertain pixels were discarded so as to only consider pixels belonging to paint/pigment layers. The evaluation was done through two metrics: sensitivity (true positive rate) and specificity (true negative rate).

### Online content

Any other methods, supplementary notes, supplementary discussion, additional references, extended data, supplementary information, and supplementary figures and tables are available in the Supplementary Materials.

## Supplementary Materials

### The PDF file includes:

Supplementary Text Figs. S1 to S6 Legends for tables S1, S3 and S4 Tables S2, S5 to S7 References

## References

[1] X. Ye, Y. Chen, L. Peng, X. Yang, Y. Bai, Application of spectroscopy technique in cultural heritage: Systematic review and bibliometric analysis. npj Herit. Sci. 13, 169 (2025).

[2] S. Dallongeville, N. Garnier, C. Rolando, C. Tokarski, Proteins in art, archaeology, and paleontology: From detection to identification. Chem. Rev. 116, 2–79 (2016). 26709533 10.1021/acs.chemrev.5b00037

[3] F. Galluzzi, S. Chaignepain, J. Arslanoglu, C. Tokarski, Hydrogen-deuterium exchange mass spectrometry to study interactions and conformational changes of proteins in paints. Biophys. Chem. 289, 106861 (2022). 35940022 10.1016/j.bpc.2022.106861

[4] A. Lluveras-Tenorio, S. Orsini, S. Pizzimenti, S. Del Seppia, M. P. Colombini, C. Duce, I. Bonaduce, Development of a GC-MS strategy for the determination of cross-linked proteins in 20th century paint tubes. Microchem. J. 170, 106633 (2021).

[5] M. Cotte, J. Susini, J. Dik, K. Janssens, Synchrotron-based X-ray absorption spectroscopy for art conservation: Looking back and looking forward. Acc. Chem. Res. 43, 705–714 (2010). 20058906 10.1021/ar900199m

[6] M. Alfeld, L. de Viguerie, Recent developments in spectroscopic imaging techniques for historical paintings—A review. Spectrochim. Acta B At. Spectrosc. 136, 81–105 (2017).

[7] L. Bertrand, M. Thoury, P. Gueriau, É. Anheim, S. Cohen, Deciphering the chemistry of cultural heritage: Targeting material properties by coupling spectral imaging with image analysis. Acc. Chem. Res. 54, 2823–2832 (2021). 34143613 10.1021/acs.accounts.1c00063

[8] P. Kret, A. Bodzon-Kulakowska, A. Drabik, J. Ner-Kluza, P. Suder, M. Smoluch, Mass spectrometry imaging of biomaterials. Materials (Basel) 16, 6343 (2023). 37763619 10.3390/ma16186343 PMC10534324

[9] M. R. L. Paine, P. C. Kooijman, G. L. Fisher, R. M. A. Heeren, F. M. Fernández, S. R. Ellis, Visualizing molecular distributions for biomaterials applications with mass spectrometry imaging: A review. J. Mater. Chem. B 5, 7444–7460 (2017). 32264222 10.1039/c7tb01100h

[10] K. Keune, J. J. Boon, Imaging secondary ion mass spectrometry of a paint cross section taken from an early netherlandish painting by Rogier van der Weyden. Anal. Chem. 76, 1374–1385 (2004). 14987095 10.1021/ac035201a

[11] J. Sanyova, S. Cersoy, P. Richardin, O. Laprévote, P. Walter, A. Brunelle, Unexpected materials in a rembrandt painting characterized by high spatial resolution cluster-TOF-SIMS imaging. Anal. Chem. 83, 753–760 (2011). 21218778 10.1021/ac1017748

[12] C. Bouvier, S. Van Nuffel, P. Walter, A. Brunelle, Time-of-flight secondary ion mass spectrometry imaging in cultural heritage: A focus on old paintings. J. Mass Spectrom. 57, e4803 (2022). 34997666 10.1002/jms.4803

[13] M. Tuck, L. Blanc, R. Touti, N. H. Patterson, S. Van Nuffel, S. Villette, J.-C. Taveau, A. Römpp, A. Brunelle, S. Lecomte, N. Desbenoit, Multimodal imaging based on vibrational spectroscopies and mass spectrometry imaging applied to biological tissue: A multiscale and multiomics review. Anal. Chem. 93, 445–477 (2021). 33253546 10.1021/acs.analchem.0c04595

[14] P. Chaurand, *MALDI Mass Spectrometry Imaging: From Fundamentals to Spatial Omics*, T. Porta Siegel, Ed. (The Royal Society of Chemistry, 2021). 10.1007/s00216-022-04127-y PMC9234028 35610380

[15] J. L. Moore, N. H. Patterson, J. L. Norris, R. M. Caprioli, Prospective on imaging mass spectrometry in clinical diagnostics. Mol. Cell. Proteomics 22, 100576 (2023). 37209813 10.1016/j.mcpro.2023.100576 PMC10545939

[16] K. K. Krestensen, R. M. A. Heeren, B. Balluff, State-of-the-art mass spectrometry imaging applications in biomedical research. Analyst 148, 6161–6187 (2023). 37947390 10.1039/d3an01495a

[17] P. Bourceau, B. Geier, V. Suerdieck, T. Bien, J. Soltwisch, K. Dreisewerd, M. Liebeke, Visualization of metabolites and microbes at high spatial resolution using MALDI mass spectrometry imaging and in situ fluorescence labeling. Nat. Protoc. 18, 3050–3079 (2023). 37674095 10.1038/s41596-023-00864-1

[18] A. Alvarez-Martin, J. Quanico, T. Scovacricchi, E. Avranovich Clerici, G. Baggerman, K. Janssens, Chemical mapping of the degradation of geranium lake in paint cross sections by MALDI-MSI. Anal. Chem. 95, 18215–18223 (2023). 37994904 10.1021/acs.analchem.3c03992

[19] V. Krupicka, F. Grelard, L. Blanc, N. Desbenoit, J. Arslanoglu, C. Tokarski, “Paint cross-section layer composition identification and prediction using MALDI-MSI” in *TECHNART2023: Non-Destructive and Microanalytical Techniques in Art and Cultural Heritage. Book of Abstracts*, M. Manso, V. Antunes, M. L. Carvalho, Eds. (Universidade Nova de Lisboa–Faculdade de Ciências e Tecnologia, Lisboa, 2023).

[20] A. Palmer, P. Phapale, I. Chernyavsky, R. Lavigne, D. Fay, A. Tarasov, V. Kovalev, J. Fuchser, S. Nikolenko, C. Pineau, M. Becker, T. Alexandrov, FDR-controlled metabolite annotation for high-resolution imaging mass spectrometry. Nat. Methods 14, 57–60 (2017). 27842059 10.1038/nmeth.4072

[21] L. Blanc, G. B. Ferraro, M. Tuck, B. Prideaux, V. Dartois, R. K. Jain, N. Desbenoit, Kendrick mass defect variation to decipher isotopic labeling in brain metastases studied by mass spectrometry imaging. Anal. Chem. 93, 16314–16319 (2021). 34860501 10.1021/acs.analchem.1c03916 PMC9841243

[22] L. Aimo, R. Liechti, N. Hyka-Nouspikel, A. Niknejad, A. Gleizes, L. Götz, D. Kuznetsov, F. P. A. David, F. G. van der Goot, H. Riezman, L. Bougueleret, I. Xenarios, A. Bridge, The SwissLipids knowledgebase for lipid biology. Bioinformatics 31, 2860–2866 (2015). 25943471 10.1093/bioinformatics/btv285 PMC4547616

[23] D. S. Wishart, Y. D. Feunang, A. Marcu, A. C. Guo, K. Liang, R. Vázquez-Fresno, T. Sajed, D. Johnson, C. Li, N. Karu, Z. Sayeeda, E. Lo, N. Assempour, M. Berjanskii, S. Singhal, D. Arndt, Y. Liang, H. Badran, J. Grant, A. Serra-Cayuela, Y. Liu, R. Mandal, V. Neveu, A. Pon, C. Knox, M. Wilson, C. Manach, A. Scalbert, HMDB 4.0: The human metabolome database for 2018. Nucleic Acids Res 46, D608–D617 (2018). 29140435 10.1093/nar/gkx1089 PMC5753273

[24] L. S. Eberlin, K. Margulis, I. Planell-Mendez, R. N. Zare, R. Tibshirani, T. A. Longacre, M. Jalali, J. A. Norton, G. A. Poultsides, Pancreatic cancer surgical resection margins: Molecular assessment by mass spectrometry imaging. PLOS Med. 13, e1002108 (2016). 27575375 10.1371/journal.pmed.1002108 PMC5019340

[25] S. Vahur, A. Teearu, T. Haljasorg, P. Burk, I. Leito, I. Kaljurand, Analysis of dammar resin with MALDI-FT-ICR-MS and APCI-FT-ICR-MS. J. Mass Spectrom. 47, 392–409 (2012). 22431467 10.1002/jms.2971

[26] D. Scalarone, M. C. Duursma, J. J. Boon, O. Chiantore, MALDI-TOF mass spectrometry on cellulosic surfaces of fresh and photo-aged di- and triterpenoid varnish resins. J. Mass Spectrom. 40, 1527–1535 (2005). 16320298 10.1002/jms.893

[27] J. L. Lazarte Luna, D. Mahon, S. A. Centeno, F. Caro, L. Smieska, “Old world, new world: Painting practices in the reformed 1686 Painter’s Guild of Mexico City” in *AIC Paintings Specialty Group Postprints* (AIC, 2018), pp. 67–74.

[28] S. A. Centeno, D. Mahon, F. Caro, J. L. Lazarte Luna, “New light on the use of ash in the ground preparations of baroque paintings from Spain, North and South America” in *Ground Layers in European Painting 1550–1750, CATS Proceedings*, A. Haack Christensen, A. Jager, J. H. Townsend Eds. (Archetype Publications Ltd., 2019), pp. 21–30.

[29] F. Grélard, D. Legland, M. Fanuel, B. Arnaud, L. Foucat, H. Rogniaux, Esmraldi: Efficient methods for the fusion of mass spectrometry and magnetic resonance images. BMC Bioinformatics 22, 56 (2021). 33557761 10.1186/s12859-020-03954-z PMC7869484

[30] F. Pozzi, J. Arslanoglu, A. Cesaratto, M. Skopek, How do you say “Bocour” in French? The work of Carmen Herrera and acrylic paints in post-war Europe. J. Cult. Herit. 35, 209–217 (2019).

[31] C. Calvano, I. van der Werf, F. Palmisano, L. Sabbatini, Fingerprinting of egg and oil binders in painted artworks by matrix-assisted laser desorption ionization time-of-flight mass spectrometry analysis of lipid oxidation by-products. Anal. Bioanal. Chem. 400, 2229–2240 (2011). 21491111 10.1007/s00216-011-4919-1

[32] I. D. van der Werf, C. D. Calvano, F. Palmisano, L. Sabbatini, A simple protocol for matrix assisted laser desorption ionization–time of flight-mass spectrometry (MALDI-TOF-MS) analysis of lipids and proteins in single microsamples of paintings. Anal. Chim. Acta 718, 1–10 (2012). 22305892 10.1016/j.aca.2011.12.056

[33] E. G. Bligh, W. J. Dyer, A rapid method of total lipid extraction and purification. Can. J. Biochem. Physiol. 37, 911–917 (1959). 13671378 10.1139/o59-099

[34] J. Erde, R. R. O. Loo, J. A. Loo, Enhanced FASP (eFASP) to increase proteome coverage and sample recovery for quantitative proteomic experiments. J. Proteome Res. 13, 1885–1895 (2014). 24552128 10.1021/pr4010019 PMC3993969

[35] F. Pozzi, J. Arslanoglu, F. Galluzzi, C. Tokarski, R. Snyder, Mixing, dipping, and fixing: The experimental drawing techniques of Thomas Gainsborough. Herit. Sci. 8, 85 (2020).

[36] F. Carò, S. A. Centeno, D. Mahon, Painting with recycled materials: On the morphology of calcite pseudomorphs as evidence of the use of wood ash residues in Baroque paintings. Herit. Sci. 6, 3 (2018).

[37] N. Garnier, C. Rolando, J. M. Høtje, C. Tokarski, Analysis of archaeological triacylglycerols by high resolution nanoESI, FT-ICR MS and IRMPD MS/MS: Application to 5th century BC–4th century AD oil lamps from Olbia (Ukraine). Int. J. Mass Spectrom. 284, 47–56 (2009).

