# Supplementary material for: Layer-by-layer decoding of contemporary and historic painting composition using MALDI mass spectrometry imaging and machine learning
Source: Sci Adv. 2026 Mar 25;12(13):eadz4427. doi: 10.1126/sciadv.adz4427 (PMC13015881; doi:10.1126/sciadv.adz4427)
Supplement: Supplementary file 1 — Supplementary Text Figs. S1 to S6 Legends for tables S1, S3 and S4 Tables S2, S5 to S7 References [file sciadv.adz4427_sm.pdf]

Science Advances
AAAS

# Supplementary Materials for

## Layer-by-layer decoding of contemporary and historic painting composition using MALDI mass spectrometry imaging and machine learning

Václav Krupička *et al.*

Corresponding author: Caroline Tokarski, caroline.tokarski@u-bordeaux.fr

*Sci. Adv.* **12**, eadz4427 (2026)
DOI: 10.1126/sciadv.adz4427

**The PDF file includes:**

Supplementary Text
Figs. S1 to S6
Legends for tables S1, S3 and S4
Tables S2, S5 to S7
References

**Other Supplementary Material for this manuscript includes the following:**

Tables S1, S3 and S4

## I- SUPPLEMENTARY NOTES

### Structure of paint cross sections from easel paintings

Cross sections taken from paintings provide a sidelong view of the layers applied to a support. Usually, these stratigraphies contain several distinct layers as seen in Fig. S6. If the complete stratigraphy of the painting is captured in a sample, it may contain the following layers.

- Support: The surface of a material on which a painting was created. In historic easel paintings, a support would have been made from canvas, wood, metal or stone.
- Preparation: Over the support, a smooth preparatory layer(s) is often applied, which can contain more than one layer, termed ground or priming depending on their function.
- Pictorial layer: Over the ground/priming, subsequent layers of paint are applied, comprised of powdered pigments mixed with a binding material. Artists may employ multiple paint layers to achieve desired artistic effects. In some instances, paintings can also have decorative appliques of metal leaf or powdered metal paint.
- Coating: The outermost layer can be a varnish, a coating applied to protect the artwork.

### Structure and composition of paint cross sections from Pratt replica panels

The Pratt paint replicas were prepared in 2008 for research purposes and consist of seven wooden boards with 36 painted squares per board of various pigments/binder combinations. All pigments, animal glue, and casein (prepared with ammonia) binders were sourced from Kremer Pigments Inc. The whole egg binder (egg tempera) was made fresh from a store-bought egg. In the replicas the ground layer consists of a mixture of animal glue and gesso (a mixture of chalk (calcium carbonate; $CaCO_3$) and gypsum (calcium sulphate dihydrate; $CaSO_4•2H_2O$), confirmed by SEM-EDS). In technical art history, the term gesso is understood as the Italian name for a specific compound, calcium sulphate dihydrate, known in English as gypsum. However, in vernacular usage, gesso has been used generically to describe any white preparatory layer, including gypsum and chalk mixtures. In this article gesso refers to a mixture of $CaCO_3$ and $CaSO_4•2H_2O$ or assignments made by the MSIpredictArt model.

The pigments and binders used to make these replicas are contemporary. This means that the binders and pigments are either purified from the natural material (milk, collagen extracted from animal tissues, earths or clays) or chemically synthesized so that the binder or colorant is composed of a few or a single compound and may be of higher purity than a historic pigment from a geological source. Nonetheless, the pigments and binders for the replicas are referred to by their traditional artistic name. They are not single chemical component materials, yet they usually have a major chemical component. Table S2 describes the composition of the artists' pigments and binders used to make the replicas.

## II- SUPPLEMENTARY METHODS

### Attenuated Total Reflection-Fourier Transform Infrared Micro-spectroscopy (m-ATR-FTIR)

The binding media within a cross section from the painting were analyzed by Attenuated Total Reflection-Fourier Transform Infrared micro-spectroscopy (m-ATR-FTIR) with a Hyperion 3000 Microscope, interfaced to a Tensor 27 spectrometer (Bruker Optics), and equipped with an MCT detector (mercury cadmium telluride), cooled with liquid nitrogen. A 20x ATR objective featuring a germanium crystal was used for measurements in contact mode. The ATR-FTIR spectra were acquired as a sum of 256 scans in the range 4000 to 550 $cm^{-1}$ and 4 $cm^{-1}$ resolution. Spectra were interpreted by comparison with the NIST 2007 library and personal databases, as well as published literature.

### Pyrolysis Gas Chromatography/Mass Spectrometry (Py-GC/MS)

Py-GC/MS analysis of *The Marriage of the Virgin* was carried out on an Agilent 8890 GC, 5977B MSD and a Frontier lab Double-Shot 3030D pyrolyzer (no autosampler) as described in Pozzi et al. (*30*) with the following changes: Samples (~50-55 micrograms of the red ground (layers 1-2) and the paint (layer 3) and gilding remnants (layer 4-5) were pyrolyzed at 550°C for 20 seconds without and with derivatization using tetramethylammonium hydroxide (TMAH; 2 µL of 25% in methanol (Fisher Scientific)) and injected on the column with a 20:1 split. The GC oven temperature program was held at 320°C for 15 min after the gradient. Data analysis was performed on an Agilent MSD ChemStation F.01.03.2357 software by comparison with the NIST 2017 spectral libraries.

### LC-MS/MS proteomics

*Materials*
Acetonitrile, ammonium bicarbonate, chloroform, deoxycholic acid, dithiothreitol, ethyl acetate, formic acid, iodoacetamide, methanol, sodium dodecyl sulfate, trifluoroacetic acid, Tween 20, and urea were purchased from Sigma (St. Louis, MO). Trypsin was purchased from Promega (Madison, WI). A Milli-Q Ultrapure deionized water purification system [resistivity 18.2 MΩ·cm (25°C)] (Millipore, Bedford, MA) was used.

*Protein and Lipid Extraction*
The painting sample was homogenized using the Bead Ruptor Elite (Omni, Kennesaw, Georgia) using 5 rounds of 4 m/s for 30 s with 4-min cooling intervals between rounds in the initial reagent. A Bligh and Dyer extraction(*31-33*) was used with 1:2 $CHCl_3$/MeOH and 20 min of sonication, the addition of another part $CHCl_3$ and 20 min sonication, then finally the addition of one part water with 20 min sonication. Centrifugation for 15 min was used to separate the layers and the bottom organic layer was removed, dried under nitrogen stream, and reserved for lipidomics analysis. Any proteins precipitated at the interface or solid pellets formed from pigments, fillers, etc. were kept with the aqueous fractions to undergo further protein extraction.

Both the lipidic and proteinaceous fractions were dried at ambient temperature in a speed vacuum system. A modified enhanced filter-aided digestion sample preparation (eFASP) based on previously described methods (*34*, *35*) was used to extract proteins and perform enzymatic hydrolysis directly on samples or on dried delipidated aqueous fractions. A buffer of 4% sodium dodecyl sulfate (SDS), 8 M urea, 0.2% deoxycholic acid (DCA), 50 mM dithiothreitol (DTT), and 100 mM ammonium bicarbonate (ABC) (pH 8.8) was used to extract proteins at 10°C overnight.

*Enzymatic Hydrolysis of Proteins*

Extracted proteins were transferred to 10 kDa Amicon Ultra-0.5 centrifugal filter units (Merck Millipore, Cork, Ireland) which were preconditioned with 5% v/v Tween 20 overnight then thoroughly rinsed. Buffer was exchanged with 8 M urea, 0.2% DCA, in 100 mM ABC (pH 8.8) using centrifugation 30 min at 13,300×*g*. Alkylation was performed using 55 mM iodoacetamide for 1 hour in the dark. Buffer was then exchanged with 0.2% DCA in 50 mM ABC (pH 8.8) and 0.1 to 0.5 µg of trypsin was added for digestion overnight at 37°C. The digested peptides were collected into a clean Amicon collection unit by centrifugation for 15 min at 13,300×*g*, repeating twice with the addition of 50 µL of 50 mM ABC to the filter. Samples were purified using a liquid-liquid extraction with the addition of ethyl acetate as well as trifluoroacetic acid (TFA) for acidification, followed by centrifugation to separate the layers then removal of the organic layer. Two subsequent washes with ethyl acetate were performed followed by evaporation of any remaining organic phase using a 60°C for 5 min. The aqueous phase was dried using a speed vacuum system, then reconstituted in minimal volumes of 0.1% formic acid for MS analysis.

*NanoLC-MS/MS*

The peptide mixture was analyzed using a Vanquish Neo nanoLC system (ThermoFisher Scientific) coupled to the Orbitrap Eclipse MS where 1 µL of sample was directly injected onto a PepMap Neo column (75 µm × 500 mm, C18, 2 µm, 100 Å, ThermoFisher Scientific), using the fast-loading injection method with Combined Control using the following gradient at a flow rate of 0.3 µL/min: 0-93 min increase from 4 to 40% B; 93-94 min, increase to 90% B; 94 – 98 min, hold at 90% B, where Buffer A was 0.1% formic acid and Buffer B was 20% water/80% acetonitrile with 0.1% formic acid. MS1 scans were recorded in the *m/z* 375–1500 range and MS2 were collected in the Orbitrap using the Normal mass mode. Data were acquired using Xcalibur 4.2 software, using a data dependent analysis (DDA) Top20 method, including the following MS2 filters: MIPS (Peptide), intensity (1e2), charge state (+2 to 7), dynamic exclusion (30s). High-energy collisional dissociation (HCD) fragmentation for MS2 was performed at 28%, using the automated injection time mode. Reference samples, blanks, as well as positive and negative controls were injected and tested alongside the samples to prevent contamination and carryover.

*Bioinformatics Analysis*

NanoLC-MS/MS raw data files were searched using PEAKS Studio X Pro (Bioinformatics Solutions Inc., Ontario, Canada) against NCBI Magnoliopsida database downloaded October

20th, 2022, and all Uniprot downloaded January 10, 2024. The protein database for flax was downloaded from Phytozome on May 15th, 2023, based on the genome *Linum usitatissimum* v1.0. The bioinformatics analysis parameters were as follows: 3 maximum missed cleavages; precursor mass error tolerance 10.0 ppm; fragment mass error tolerance 0.02 Da. PEAKS post translational modification (PTM) algorithm was used to identify common fixed modifications (carbamidomethylation) and variable modifications [i.e. oxidation (methionine, proline), deamidation (asparagine, glutamine), acetylation (N-terminus)]. The PEAKS SPIDER algorithm was used to identify amino acid modification and search for cross-species homology. The protein score threshold of -10lgP ≥ 20 and a peptide false discovery rate (FDR) of 0.1%. New peptide sequences were characterized de novo using the same score restrictions to expand the databases in house. At least two different non-overlapping peptides were required for protein identification. MS2 spectra of peptides were inspected manually, and the NCBI database and Phytozome Basic Local Alignment Search Tools (BLAST) were used to verify species specificity.

## IV- SUPPLEMENTARY DISCUSSION

### Historic sample from *The Marriage of the Virgin* by José Sanchez

In 2016, The Metropolitan Museum of Art (The Met) acquired *The Marriage of the Virgin* by José Sanchez, a Mexican artist active between 1686 and 1695. *The Marriage* was originally part of a large altarpiece depicting the life of the Virgin Mary. This painting along with *The Visitation* at the Musée du Louvre are the only two works from the altarpiece currently in public collections.

### Results for complementary analysis of the historic sample from *The Marriage of the Virgin* by José Sanchez

A cross section from a sample obtained from the proper left edge of the gilded trompe-l'œil frame was analyzed. This cross section contains six distinct layers as shown in Fig. 3. Two red grounds (layers 1 and 2) compose the preparatory layer over the bast-fiber canvas support. Over the red grounds, two paint layers can be observed, a pink paint (layer 3) and a light brown paint (layer 4). On the top of the light brown paint is a fine layer of gold leaf (layer 5) adhered using an oil size. The oil size may have sunk into the lower paint layers as it no longer appears as a distinct layer. At some point in the late 19$^{th}$ century the painting was conserved and relined, which entailed the adhesion of a secondary canvas support to the verso using a wheat paste adhesive. As customary in treatments from the period, the lining was activated using pressure and heat from irons which can cause the infusion of the lining adhesive into the paint layers. *The Marriage* was re-stretched onto a wooden stretcher most likely using a system comparable to the "Dutch method" in which paper strips (layer 6) were adhered to the edges of the painting's recto to tension it onto a large strainer and mitigate planar deformation upon drying. As described in the main text, the inorganic pigments of the Sanchez painting have been previously studied by SEM-EDS and Raman spectroscopy (27, 28, 36) Sanchez's technique is in line with those found in paintings by other Mexican artists from the late seventeenth century. The use of double grounds containing red earth (hematite) and plant-ash is a tradition that central Mexico shared with Madrid and one that has been studied since 2018 (*36*). The composition of the ground and paint layers was previously characterized by SEM-EDS and Raman spectroscopy (*27, 28*), establishing the presence of red earth as well as calcite pseudomorphs and associated carbon black originating from plant ash in ground layer 1, with ground layer 2 being predominantly composed of the red earth. The pink paint layer was not previously studied but observation by optical microscopy suggests that it is composed of an organic red lake dye precipitated onto an inert substrate. The light brown paint used by José Sanchez contained lead white pigment as identified by SEM-EDS with gold leaf adhered over it likely using a dark pigmented oil sizing. The oil sizing may have sunk into the lower light brown paint layer (layer 4), as it no longer appears as a distinct layer. In 1686, Sanchez was chosen as an inspector in the reformed Guild for Painters and Gilders of Mexico City. In this capacity had great influence and oversaw the work of seasoned painters hoping to establish their own workshop practices. Since Sanchez was

establishing modes of practice in the city, the study of his materials and techniques is crucial to the contextualization of regional artistic practice in the late 17$^{th}$ century. Aside from the pigments discussed in *The Marriage* sample above, Sanchez made use of a variety of pigments common in the palette of the seventeenth century, such as, lead white, smalt, vermillion, orpiment, ochres, verdigris, and earth pigments.

The binder composition of individual layers was studied using m-ATR-FTIR, Py-GC/MS, and LC-MS/MS. Binding media analysis by ATR-FTIR was not conclusive as the spectra were dominated by the inorganic components. The light brown layer (layer 4) contained peaks at 2920 and 2851 $cm^{-1}$ indicating a possible oil binder. All layers could contain protein (1651 and 1553 $cm^{-1}$) but the source could be the conservation adhesive (bovine glue) used during the lining treatment. Binding medium analysis by Py-GC/MS with and without TMAH revealed that the red ground layers (layers 1 and 2 combined) and the paint layer with gilding remnants contained protein, oil, and pine resin. Pine resin was identified by TMAH Py-GC/MS through methylated abietic acid degradation products: methyl dehydroabietate, 7-hydroxy-8,11,13-abietatrien-19-oic acid, 2Me derivative, and tetradehydroabietic acid, 7-methoxy-, methyl ester. Protein was identified through pyrrole, diketopyrroles (*m/z* 70 and 154 pyrrolo[1,2-a]pyrazine-1,4-dione, hexahydro-) and pyrrolidinone adducts. The profile is most consistent with collagen protein. No cholesterol was identified, eliminating the presence of egg yolk. Oil was identified by the presence of cyclic fatty acids in Py-GC/MS and methylated glycerol and fatty acids in TMAH Py-GC/MS. Fatty acid ratios of palmitic/stearic acid (P/S) and azelaic/palmitic acid (A/P) shown in Supplementary Table 6 do not correlate to a drying oil (A/P<1) such as linseed, walnut, or poppy oil.[1] Furthermore, the fatty acid profile excludes the addition of wax or other possible traditional Western European semi-drying or non-drying oils (sunflower, safflower, rapeseed, castor, tung, or fish oils) that could be mixed with linseed oil. The ratios of the diacids suberic and sebacic acids to azelaic acid (Sub/A and Seb/Az) are not elevated indicating that the oil was not prepolymerized. While the low A/P ratios may suggest the presence of aged egg yolk, egg was not identified in the painting by bottom-up proteomics using LC-MS/MS and therefore a non-drying oil binder was used by the artist. The red grounds contain relatively more collagen protein than oil, and the paint contains relatively more oil than collagen protein by comparison of the peak intensities for similar sample masses. The two grounds (layers 1 and 2) could not be separated for Py-GC/MS analysis. The results may suggest that one ground layer contains a proteinaceous binder while the other is oil-based. Alternatively, the presence of collagen protein could be attributed to the conservation lining treatment. The paint binder appears to be oil, and—as previously noted—the collagen protein may result from conservation intervention. The presence of pine resin is higher in the paint layer, most likely indicating a pine resin varnish coating. The presence of pine resin in the ground layers is most likely due to contamination, since the sample was removed from the edge of the painting.

Elsewhere, the sample taken from *The Marriage*'s edge for MALDI-MSI was divided in two, with the remaining portion being subjected to proteomics analysis by LC-MS/MS to further characterize the proteinaceous binders. Bottom-up proteomic analyses were performed with homogenization of the bulk sample and no separation of the layers, as such, no spatial information is provided from this set of data. Prior to protein extraction, an adapted Bligh and Dyer delipidation step was performed, as described in the Supplementary Methods, and the lipidic fraction was reserved for further studies. The remaining aqueous fraction and inorganic residue underwent an enhanced filter-aided sample preparation (eFASP) to extract and digest proteins. LC-MS/MS analyses identified proteins from two proteinaceous binders, bovine animal glue and wheat starch paste, as shown in Table S7. MS2 from peptides in collagen alpha 1(III) identified the animal glue to be from cattle [*Bos taurus*]; as only one species of collagen was identified in the sample, it is probable that *Bos* animal glue was used in both the original grounds and the restoration (Dutch-method, paper layer), knowing the MSI-spatial data. Several proteins were identified from the Triticinae genus (Table S7) and MS2 from peptides in avenin a1 confirmed the wheat starch paste was prepared using common wheat [*Triticum aestivum*]. Given the conservation history of *The Marriage*, the presence of wheat proteins can be attributed to the relining of the canvas in a method similar to the "Dutch-method".

## V- SUPPLEMENTARY FIGURES

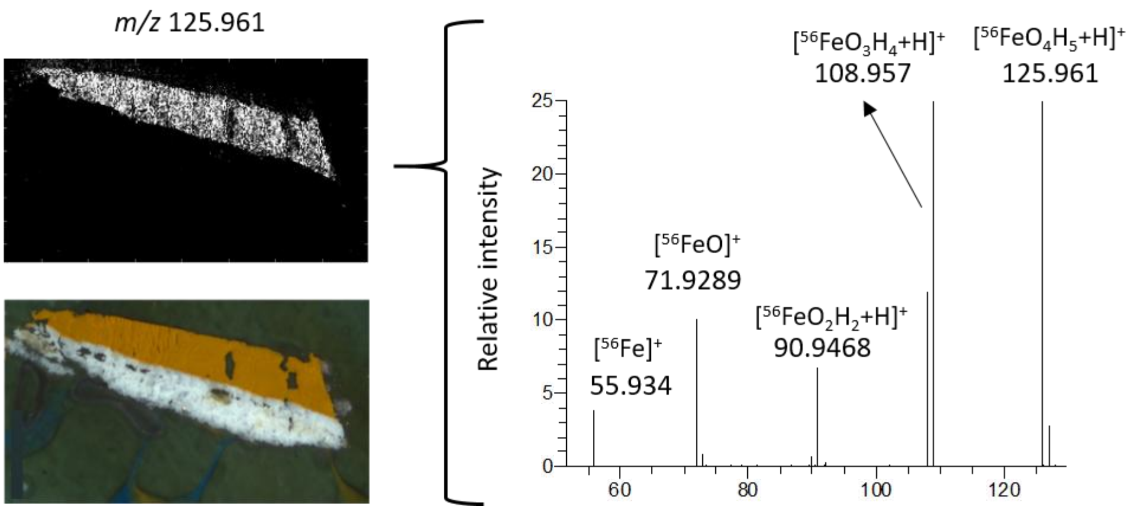


**Fig. S1. Pigment identification using MS/MS.** MS/MS of ion originating in ochre layer of P2C4 confirming the composition of the pigment as yellow ochre.

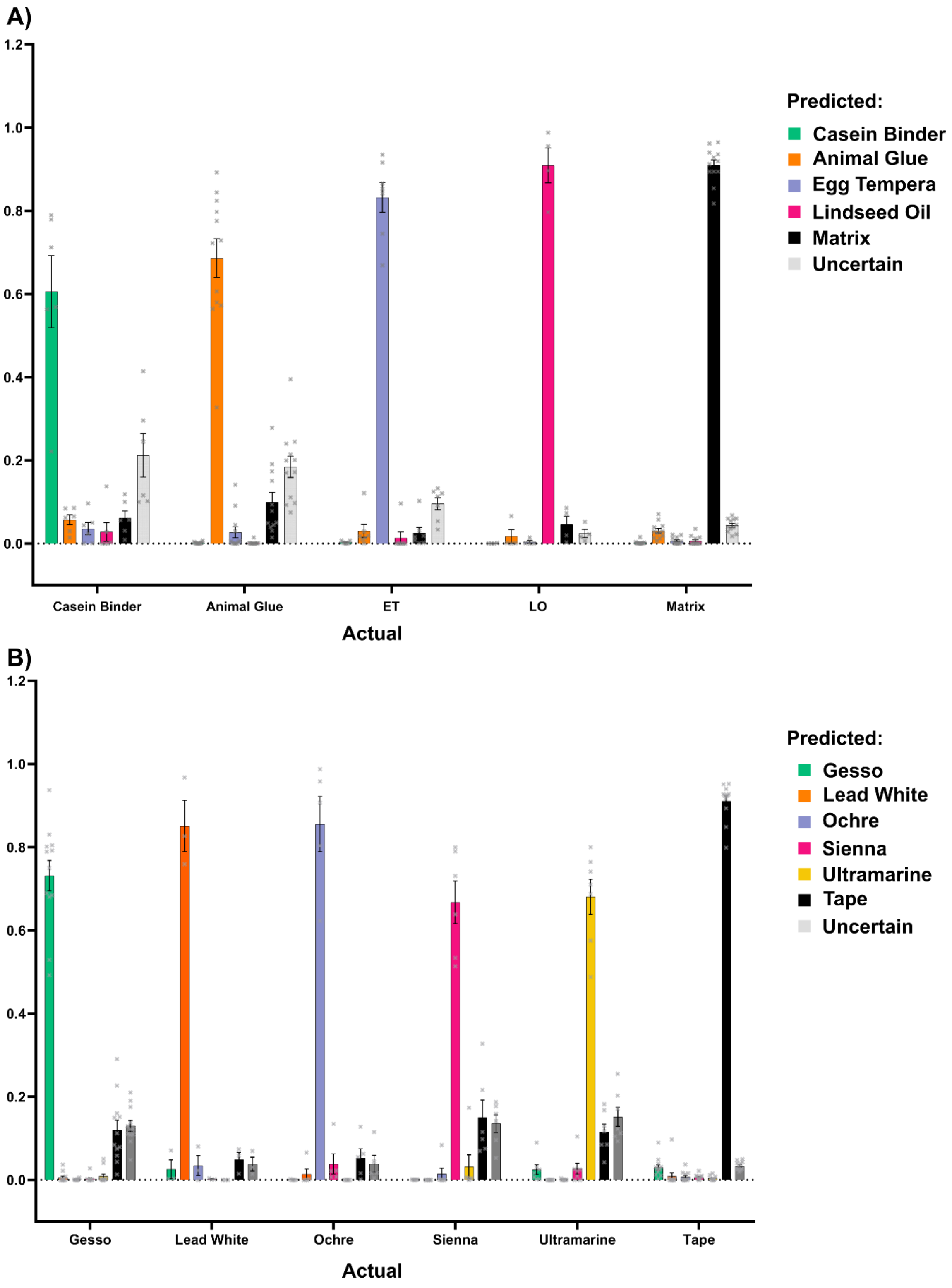


**Fig. S2. Plots representing distribution of predicted pixels for each actual class** of A) binders and B) pigments. Each data point represents a sensitivity value for a layer in a given dataset. Uncertain class is generated for pixels filtered out by the GMM clustering step.

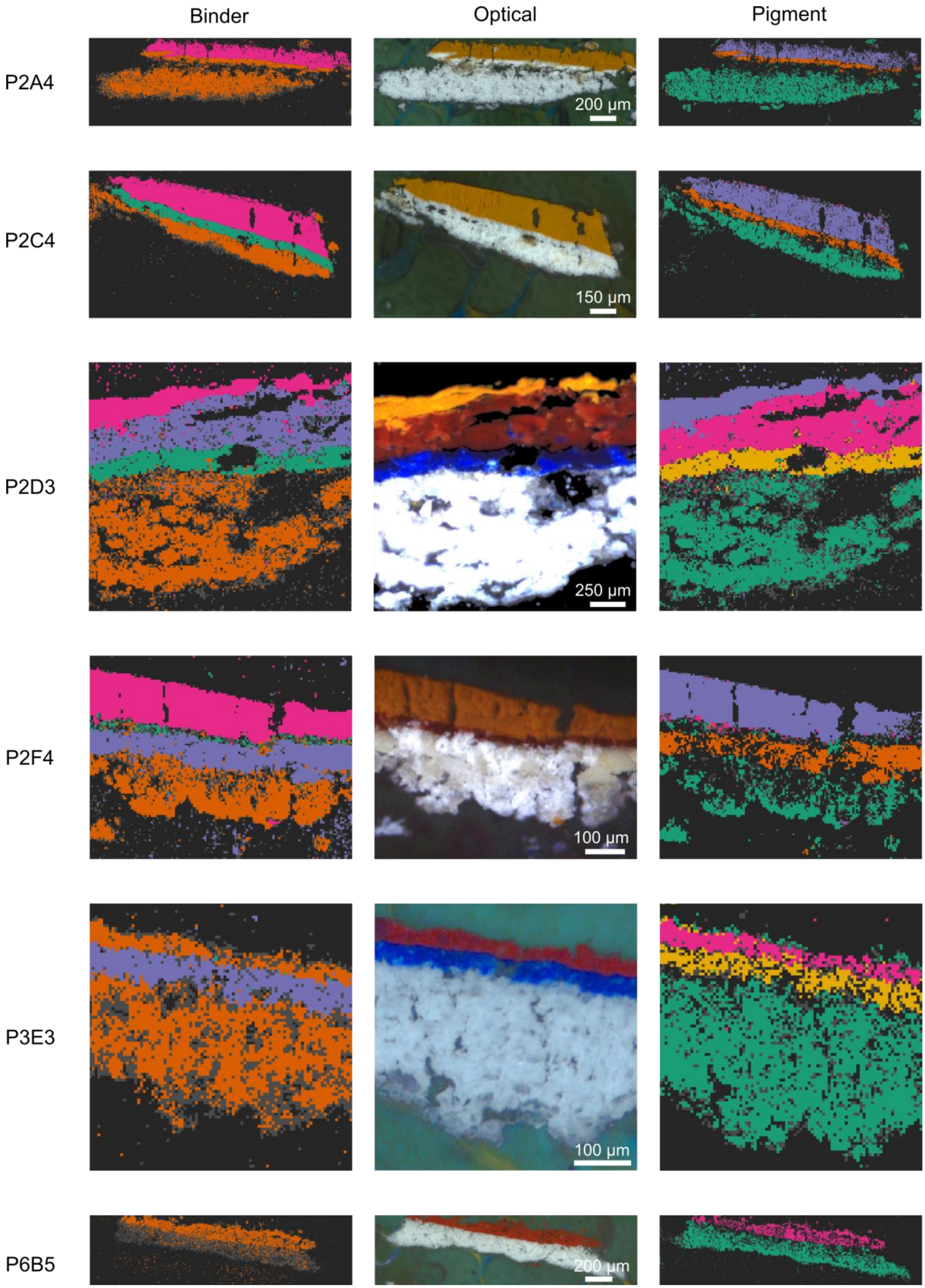

Binder
Optical
Pigment
P2A4
200 µm
P2C4
150 µm
P2D3
250 µm
P2F4
100 µm
P3E3
100 µm
P6B5
200 µm

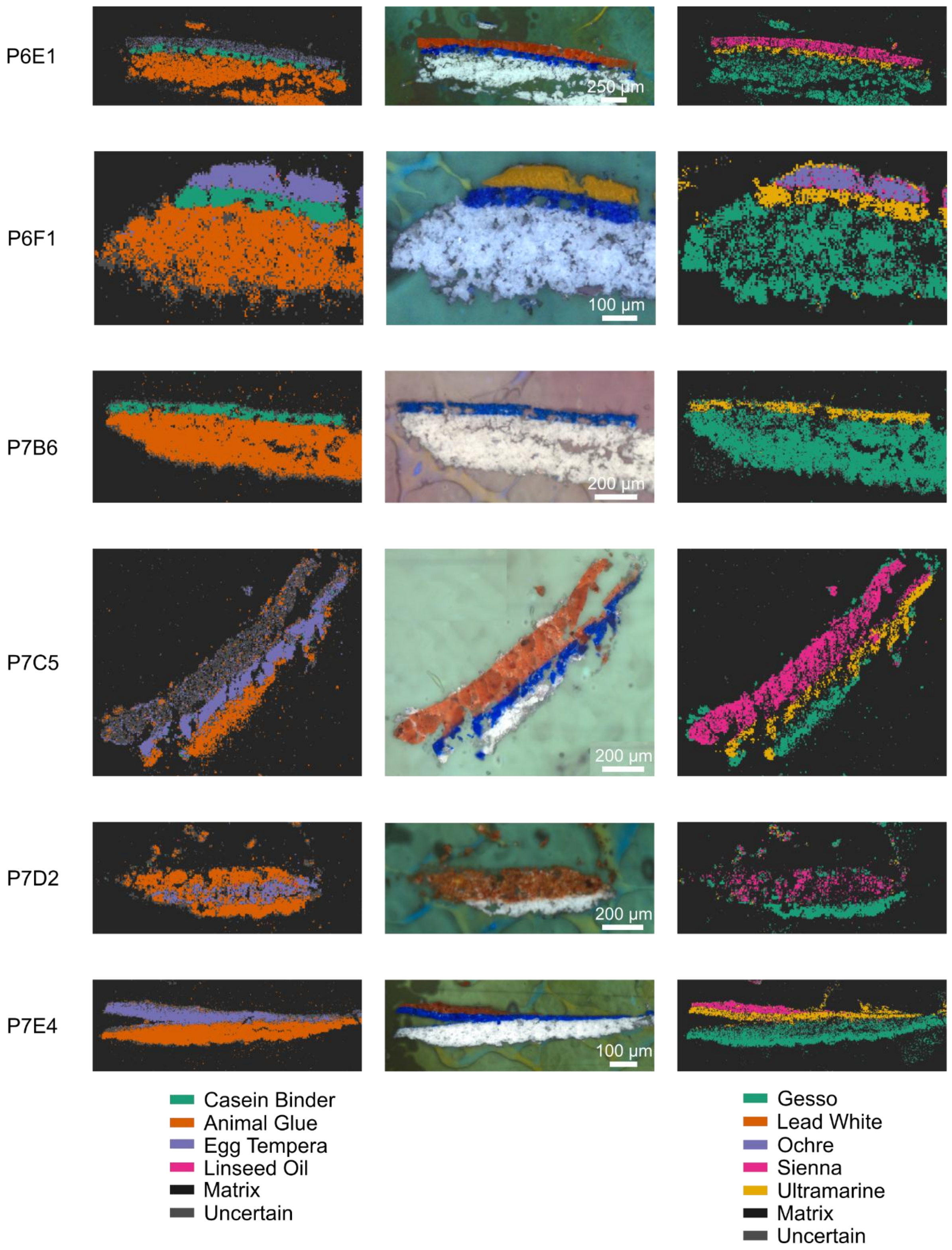


**Fig. S3. LASSO prediction for Pratt paint replicas.** Bright field microscopy images with corresponding binder and pigment LASSO predictions of paint cross sections included in the training and validation datasets for development of the LASSO predictive model.

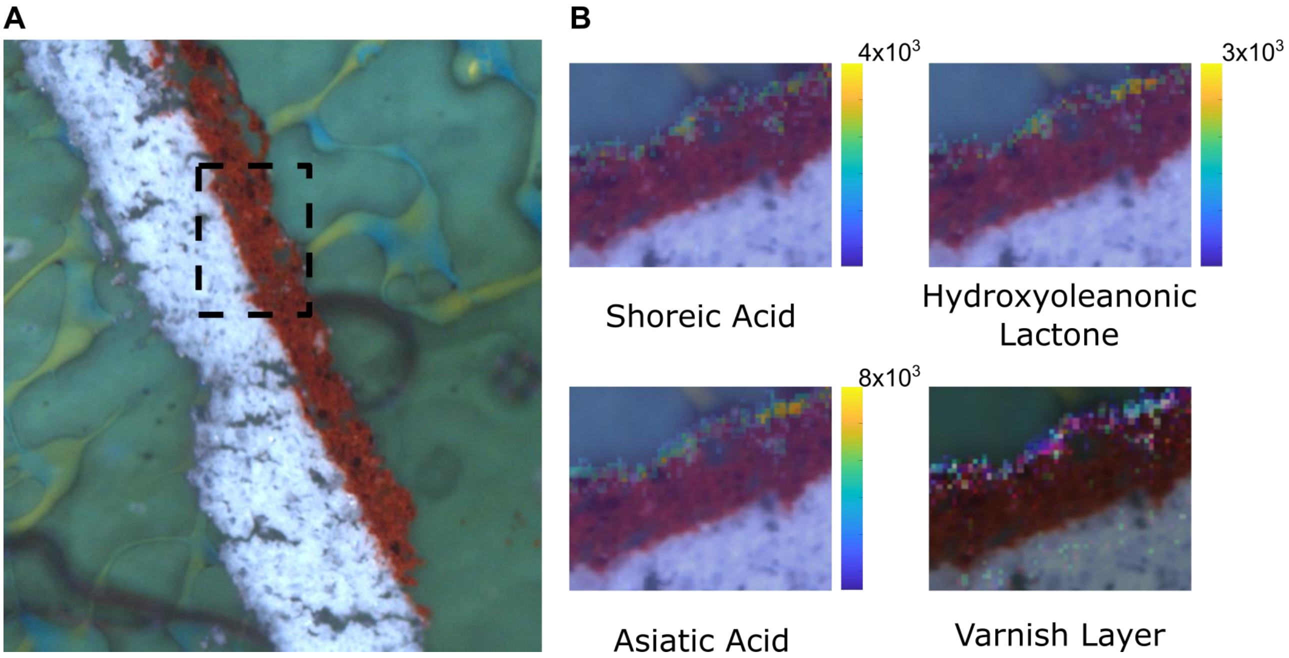


**Fig S4. Varnish layer in P3E5 dataset. (**A) Bright field images of the paint cross section for P3E5 dataset containing transparent varnish layer with highlighted ROI. (B) Extracted ion images for (clockwise from top left) shoreic acid, hydroxyoleanonic lactone, and asiatic acid, serving as biomarkers for detecting the presence of dammar varnish layer.

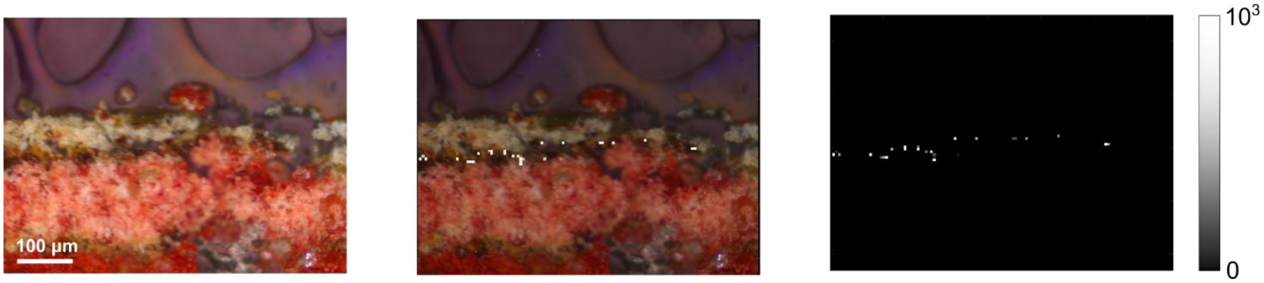


**Fig S5. Gold leaf identification in *The Marriage of the Virgin* using MSI.** Extracted ion maps for $^{197}Au$ isotope (*m/z* 196.9666) consistent with the presence of gold leaf on top of the light brown paint (layer 4). Left to right, optical image of the gold containing area, overlay of observed signal for gold isotopes with saturated intensity for ease of visualization, and extracted ion map.

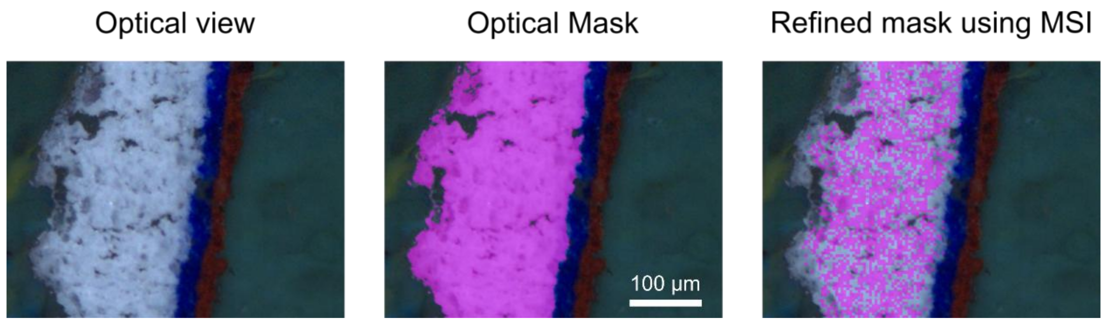


**Fig. S6. Optical mask refinement using MSI datasets in the P3E3 dataset.** Mask generated using brightfield microscopy was refined using aligned MSI datasets to obtain masks with pixels representative of the layer composition.

## VI- Supplementary Tables

**Table S1.** (separate xls file) Composition of 15-year-old multi-layer paint replicas prepared at the Pratt Institute (Brooklyn, NY, USA).

**Table S2.** Description of composition of artists pigments and binders.

| Material | Artistic Name | Composition/ Preparation | Major chemical component(s) |
|---|---|---|---|
| Pigment | Gesso | Mixture of chalk ($CaCO_3$) and gypsum ($CaSO_4•2H_2O$) | Calcium carbonate ($CaCO_3$) and calcium sulphate dihydrate ($CaSO_4•2H_2O$) |
| | Lead white | | Basic lead(II)-carbonate (2 $PbCO_3 \cdot Pb(OH)_2$) |
| | Ultramarine | A complex sulfur-containing sodium aluminum silicate. Since the 19th century, ultramarine has been manufactured artificially. | $Na_{8-10}Al_6Si_6O_{24}S_{2-4}$ |
| | Sienna | A natural clay which is partially composed of iron oxides. The most prevalent iron oxides are the iron ores limonite and goethite. In addition, natural or raw sienna is also composed of manganese oxide and aluminum oxides. | Iron(III)-oxide, partly hydrated + manganese oxide + aluminum oxide ($Fe_2O_3$(•$H_2O$) + $MnO_2$•(n $H_2O$)+ $Al_2O_3$) |
| | Umber | A natural clay which contains a mixture of iron and manganese oxides and hydroxides | Iron(III)-oxide, partly hydrated + manganese oxide + aluminum oxide ($Fe_2O_3$(•$H_2O$) + $MnO_2$•(n $H_2O$)+ $Al_2O_3$) |
| | Red or Yellow Ochre | Red = a natural clay earth pigment that is a mixture of ferric oxide and varying amounts of clay and sand. The red color is from the iron ore hematite.<br><br>Yellow = natural mineral consisting of silica and clay owing its color to an iron oxyhydroxide mineral, goethite. | Anhydrous iron(III)-oxide ($Fe_2O_3$)<br><br>Iron oxyhydroxide (FeO(OH)) |
| Binder | Animal Glue | Glue pellets prepared from proteins extracted from animal skin, bones and tendons are solubilized in simmering water. | Collagen type I |

| | | | |
|---|---|---|---|
| | Casein prepared with ammonia | Casein powder prepared from defatted milk is solubilized with ammonia. | Casein proteins |
| | Egg Tempera | Mixture of the egg yolk with a portion of the egg white. | Main proteins: ovalbumin, vitellogenin, ovotransferrin, phosvitin, lipovitellins<br>Main lipids: triglycerides, phospholipids (e.g. phosphatidylcholine, phosphatidylethanolamine), cholesterol |
| | Linseed Oil | Commercial oil extracted from the dried seeds of the flax plant (*Linum usitatissimum*) | Triglycerides (see details in (*37*))<br>Fatty acids including the saturated acids, palmitic acid and stearic acid, the monounsaturated oleic acid, and the doubly unsaturated linoleic acid. |
| | Turpentine | A fluid obtained by the distillation of resin harvested from living trees, mainly *Pinus* species. | Terpenes, primarily the monoterpenes alpha- and beta-pinene, with lesser amounts of carene, camphene, dipentene, and terpinolene |
| Varnish | Dammar | A resin obtained from the tree family *Dipterocarpaceae* in India and Southeast Asia, principally those of the genera *Shorea* or *Hopea* (synonym *Balanocarpus*). | Triterpenoid resin, containing many low molecular weight triterpenes (dammarane, dammarenolic acid, oleanane, oleanonic acid, etc.), and their oxidation products. |

**Table S3.** (separate xls file) Pigment result statistics for Pratt paint replica datasets included in model training and validation

**Table S4.** (separate xls file) Binder result statistics for Pratt paint replica datasets included in training and validation. LO refers to linseed oil, ET refers to egg tempera.

**Table S5.** Composition of individual paint layers and ground layers in paint cross section obtained from *The Marriage of the Virgin* by José Sanchez.

| Layer | Name | | MSI Result | Supplemental Analyses (Method Validation) | |
|---|---|---|---|---|---|
| | | | | **Spatially resolved techniques**[a] | **Bulk analysis techniques**[b] |
| 1 | Bottom red ground | *Pigment* | Gesso[e] | Calcite pseudomorphs and carbon,[f] red earths | Protein: collagen [*Bos taurus*] (animal glue), common wheat [*Triticum aestivum*] likely starch<br><br>Oil: unspecified<br><br>Pine resin |
| | | *Binder* | Animal Glue | Not identified | |
| 2 | Top red ground | *Pigment* | Iron oxide pigments[g] | Red earths | |
| | | *Binder* | Animal glue and linseed oil | Not identified | |
| 3 | Pink paint | *Pigment* | Not identified | Calcium sulfate Possible red lake | |
| | | *Binder* | Not identified | N/A | |
| 4 | Light Brown Paint (Oil gilding adhesive) | *Pigment* | Lead white | Lead white | |
| | | *Binder* | Not identified | Possible oil binder[h] | |
| 5[c] | Gold leaf | | Gold isotope | Gold leaf | |
| 6[d] | Paper (conservation treatment) and varnish layer | | Varnish | N/A | |

[a] Techniques used: SEM-EDS and Raman Spectroscopy, m-ATR-FTIR.

[b] Bulk analysis with limited or no manual separation of layers. Techniques used: Py-GC/MS (manual separation of grounds from paint and gilding), LC-MS/MS (no separation of layers including the paper from the conservation treatment).

[c] Gold leaf was adhered over the paint layers using an oil gilding sizing; therefore, the oil-containing layer could be considered a separate layer that soaked into the lower paint (Layer 4).

[d] Original varnish was most likely mastic resin with dammar resin used during conservation treatment after acquisition by the MET. The varnish soaked through and saturated the paper strips used during relining.

[e] In the replicas used to train the LASSO model, gesso refers to a mixture of calcium carbonate ($CaCO_3$) and gypsum ($CaSO_4 \bullet 2H_2O$). Previous analyses identified calcite ($CaCO_3$) from washed ash in this layer.

[f]Byproducts of potassium extraction from wood ash to produce lye.

[g] Iron oxides pigments (such as ochre and sienna), may be present in the naturally occurring mineral mixture known as red earth.

[h] See details in the section "Results for complementary analysis of the historic sample from The Marriage of the Virgin by José Sanchez"

**Table S6.** Fatty acids ratios of the ground layers (layers 1 and 2 combined) and paint layers from the José Sanchez painting obtained from analysis with TMAH Py-GC/MS.

| | **P/S** | **A/P** | **Seb/A** | **Sub/A** |
|---|---|---|---|---|
| Red Grounds (layers 1 and 2) | 1.33 | 0.27 | 0.17 | 0.14 |
| Paint (and remnants of gilding) | 1.64 | 0.44 | 0.06 | 0.16 |

**Table S7.** Significant Art Material Proteins Identified in Hydrolyzed Peptide Extracts from the José Sanchez *The Marriage of the Virgin* Using NanoLC-Orbitrap MS/MS with FDR 0.1%.

| **Database** | **Identified protein[a]** | **Accession** | **-10lgP** | **Sequence Coverage (%)** | **Number Peptides** | **Number Unique Peptides** | **Molecular Mass (Da)** |
|---|---|---|---|---|---|---|---|
| Magnoliopsida and *Linum usitatissimum* | alpha-amylase inhibitor 0.53 [*Triticum urartu*] | XP_048561932.1 | 117.23 | 27 | 3 | 1 | 16874 |
| | Avenin-like a3 [*Triticum aestivum*] | P0CZ08.1 | 116.75 | 28 | 4 | 1 | 19251 |
| | alpha-amylase inhibitor 0.28 [*Triticum aestivum*] | P01083.3 | 116.49 | 31 | 4 | 4 | 16800 |
| | alpha-amylase/trypsin inhibitor CM3 [*Triticum aestivum*] | P17314.1 | 110.03 | 38 | 4 | 4 | 18221 |
| | Avenin-like a5 [*Triticum aestivum*] | P0CZ09.1 | 108.39 | 20 | 3 | 1 | 18924 |
| | Alpha-amylase/trypsin inhibitor CM16 [*Triticum aestivum*] | P16159.1 | 97.46 | 28 | 3 | 3 | 15782 |
| | Alpha-amylase/trypsin inhibitor CM2 [*Triticum aestivum*] | P16851.2 | 95.76 | 32 | 3 | 3 | 15460 |
| | Non-specific lipid-transfer protein 2G [*Triticum aestivum*] | P82900.2 | 92.05 | 34 | 3 | 2 | 9832 |

| | | | | | | | |
|---|---|---|---|---|---|---|---|
| | beta-amylase [*Triticum urartu*] | XP_048533049.1 | 79.27 | 8 | 3 | 3 | 58747 |
| | Purothionin A-1 [*Triticum aestivum*] | P01543.2 | 71.22 | 12 | 2 | 1 | 14625 |
| | Serpin-Z1A [*Triticum aestivum*] | Q41593.1 | 69.58 | 5 | 2 | 2 | 43118 |
| Uniprot | Collagen alpha-1(I) chain [*Bos taurus*] | P02453 | 445.61 | 70 | 720 | 56 | 138939 |
| | Collagen alpha-2(I) chain [*Bos taurus*] | P02465 | 330.61 | 66 | 310 | 55 | 129064 |
| | Collagen alpha-1(III) chain [*Bos taurus*] | P04258 | 315.68 | 68 | 192 | 121 | 93651 |
| | Collagen alpha-1(II) chain [*Bos taurus*] | P02459 | 196.53 | 25 | 51 | 8 | 141829 |

[a]Multiple species-specific peptides identified using manual inspection of MS2 and BLAST for both *Triticum aestivum* and *Bos taurus*

## REFERENCES


1. X. Ye, Y. Chen, L. Peng, X. Yang, Y. Bai, Application of spectroscopy technique in cultural heritage: Systematic review and bibliometric analysis. *npj Herit. Sci.* **13**, 169 (2025).

2. S. Dallongeville, N. Garnier, C. Rolando, C. Tokarski, Proteins in art, archaeology, and paleontology: From detection to identification. *Chem. Rev.* **116**, 2–79 (2016).

3. F. Galluzzi, S. Chaignepain, J. Arslanoglu, C. Tokarski, Hydrogen-deuterium exchange mass spectrometry to study interactions and conformational changes of proteins in paints. *Biophys. Chem.* **289**, 106861 (2022).

4. A. Lluveras-Tenorio, S. Orsini, S. Pizzimenti, S. Del Seppia, M. P. Colombini, C. Duce, I. Bonaduce, Development of a GC-MS strategy for the determination of cross-linked proteins in 20th century paint tubes. *Microchem. J.* **170**, 106633 (2021).

5. M. Cotte, J. Susini, J. Dik, K. Janssens, Synchrotron-based X-ray absorption spectroscopy for art conservation: Looking back and looking forward. *Acc. Chem. Res.* **43**, 705–714 (2010).

6. M. Alfeld, L. de Viguerie, Recent developments in spectroscopic imaging techniques for historical paintings—A review. *Spectrochim. Acta B At. Spectrosc.* **136**, 81–105 (2017).

7. L. Bertrand, M. Thoury, P. Gueriau, É. Anheim, S. Cohen, Deciphering the chemistry of cultural heritage: Targeting material properties by coupling spectral imaging with image analysis. *Acc. Chem. Res.* **54**, 2823–2832 (2021).

8. P. Kret, A. Bodzon-Kulakowska, A. Drabik, J. Ner-Kluza, P. Suder, M. Smoluch, Mass spectrometry imaging of biomaterials. *Materials (Basel)* **16**, 6343 (2023).

9. M. R. L. Paine, P. C. Kooijman, G. L. Fisher, R. M. A. Heeren, F. M. Fernández, S. R. Ellis, Visualizing molecular distributions for biomaterials applications with mass spectrometry imaging: A review. *J. Mater. Chem. B* **5**, 7444–7460 (2017).

10. K. Keune, J. J. Boon, Imaging secondary ion mass spectrometry of a paint cross section taken from an early netherlandish painting by Rogier van der Weyden. *Anal. Chem.* **76**, 1374–1385 (2004).

11. J. Sanyova, S. Cersoy, P. Richardin, O. Laprévote, P. Walter, A. Brunelle, Unexpected materials in a rembrandt painting characterized by high spatial resolution cluster-TOF-SIMS imaging. *Anal. Chem.* **83**, 753–760 (2011).

12. C. Bouvier, S. Van Nuffel, P. Walter, A. Brunelle, Time-of-flight secondary ion mass spectrometry imaging in cultural heritage: A focus on old paintings. *J. Mass Spectrom.* **57**, e4803 (2022).

13. M. Tuck, L. Blanc, R. Touti, N. H. Patterson, S. Van Nuffel, S. Villette, J.-C. Taveau, A. Römpp, A. Brunelle, S. Lecomte, N. Desbenoit, Multimodal imaging based on vibrational spectroscopies and mass spectrometry imaging applied to biological tissue: A multiscale and multiomics review. *Anal. Chem.* **93**, 445–477 (2021).

14. P. Chaurand, *MALDI Mass Spectrometry Imaging: From Fundamentals to Spatial Omics*, T. Porta Siegel, Ed. (The Royal Society of Chemistry, 2021).

15. J. L. Moore, N. H. Patterson, J. L. Norris, R. M. Caprioli, Prospective on imaging mass spectrometry in clinical diagnostics. *Mol. Cell. Proteomics* **22**, 100576 (2023).

16. K. K. Krestensen, R. M. A. Heeren, B. Balluff, State-of-the-art mass spectrometry imaging applications in biomedical research. *Analyst* **148**, 6161–6187 (2023).

17. P. Bourceau, B. Geier, V. Suerdieck, T. Bien, J. Soltwisch, K. Dreisewerd, M. Liebeke, Visualization of metabolites and microbes at high spatial resolution using MALDI mass spectrometry imaging and in situ fluorescence labeling. *Nat. Protoc.* **18**, 3050–3079 (2023).

18. A. Alvarez-Martin, J. Quanico, T. Scovacricchi, E. Avranovich Clerici, G. Baggerman, K. Janssens, Chemical mapping of the degradation of geranium lake in paint cross sections by MALDI-MSI. *Anal. Chem.* **95**, 18215–18223 (2023).

19. V. Krupicka, F. Grelard, L. Blanc, N. Desbenoit, J. Arslanoglu, C. Tokarski, “Paint cross-section layer composition identification and prediction using MALDI-MSI” in *TECHNART2023: Non-Destructive and Microanalytical Techniques in Art and Cultural Heritage. Book of Abstracts*, M. Manso, V. Antunes, M. L. Carvalho, Eds. (Universidade Nova de Lisboa–Faculdade de Ciências e Tecnologia, Lisboa, 2023).

20. A. Palmer, P. Phapale, I. Chernyavsky, R. Lavigne, D. Fay, A. Tarasov, V. Kovalev, J. Fuchser, S. Nikolenko, C. Pineau, M. Becker, T. Alexandrov, FDR-controlled metabolite annotation for high-resolution imaging mass spectrometry. *Nat. Methods* **14**, 57–60 (2017).

21. L. Blanc, G. B. Ferraro, M. Tuck, B. Prideaux, V. Dartois, R. K. Jain, N. Desbenoit, Kendrick mass defect variation to decipher isotopic labeling in brain metastases studied by mass spectrometry imaging. *Anal. Chem.* **93**, 16314–16319 (2021).

22. L. Aimo, R. Liechti, N. Hyka-Nouspikel, A. Niknejad, A. Gleizes, L. Götz, D. Kuznetsov, F. P. A. David, F. G. van der Goot, H. Riezman, L. Bougueleret, I. Xenarios, A. Bridge, The SwissLipids knowledgebase for lipid biology. *Bioinformatics* **31**, 2860–2866 (2015).

23. D. S. Wishart, Y. D. Feunang, A. Marcu, A. C. Guo, K. Liang, R. Vázquez-Fresno, T. Sajed, D. Johnson, C. Li, N. Karu, Z. Sayeeda, E. Lo, N. Assempour, M. Berjanskii, S. Singhal, D. Arndt, Y. Liang, H. Badran, J. Grant, A. Serra-Cayuela, Y. Liu, R. Mandal, V. Neveu, A. Pon, C. Knox, M. Wilson, C. Manach, A. Scalbert, HMDB 4.0: The human metabolome database for 2018. *Nucleic Acids Res* **46**, D608–D617 (2018).

24. L. S. Eberlin, K. Margulis, I. Planell-Mendez, R. N. Zare, R. Tibshirani, T. A. Longacre, M. Jalali, J. A. Norton, G. A. Poultsides, Pancreatic cancer surgical resection margins: Molecular assessment by mass spectrometry imaging. *PLOS Med.* **13**, e1002108 (2016).

25. S. Vahur, A. Teearu, T. Haljasorg, P. Burk, I. Leito, I. Kaljurand, Analysis of dammar resin with MALDI-FT-ICR-MS and APCI-FT-ICR-MS. *J. Mass Spectrom.* **47**, 392–409 (2012).

26. D. Scalarone, M. C. Duursma, J. J. Boon, O. Chiantore, MALDI-TOF mass spectrometry on cellulosic surfaces of fresh and photo-aged di- and triterpenoid varnish resins. *J. Mass Spectrom.* **40**, 1527–1535 (2005).

27. J. L. Lazarte Luna, D. Mahon, S. A. Centeno, F. Caro, L. Smieska, “Old world, new world: Painting practices in the reformed 1686 Painter’s Guild of Mexico City” in *AIC Paintings Specialty Group Postprints* (AIC, 2018), pp. 67–74.

28. S. A. Centeno, D. Mahon, F. Caro, J. L. Lazarte Luna, “New light on the use of ash in the ground preparations of baroque paintings from Spain, North and South America” in *Ground Layers in European Painting 1550–1750, CATS Proceedings*, A. Haack Christensen, A. Jager, J. H. Townsend Eds. (Archetype Publications Ltd., 2019), pp. 21–30.

29. F. Grélard, D. Legland, M. Fanuel, B. Arnaud, L. Foucat, H. Rogniaux, Esmraldi: Efficient methods for the fusion of mass spectrometry and magnetic resonance images. *BMC Bioinformatics* **22**, 56 (2021).

30. F. Pozzi, J. Arslanoglu, A. Cesaratto, M. Skopek, How do you say “Bocour” in French? The work of Carmen Herrera and acrylic paints in post-war Europe. *J. Cult. Herit.* **35**, 209–217 (2019).

31. C. Calvano, I. van der Werf, F. Palmisano, L. Sabbatini, Fingerprinting of egg and oil binders in painted artworks by matrix-assisted laser desorption ionization time-of-flight mass spectrometry analysis of lipid oxidation by-products. *Anal. Bioanal. Chem.* **400**, 2229–2240 (2011).

32. I. D. van der Werf, C. D. Calvano, F. Palmisano, L. Sabbatini, A simple protocol for matrix assisted laser desorption ionization–time of flight-mass spectrometry (MALDI-TOF-MS) analysis of lipids and proteins in single microsamples of paintings. *Anal. Chim. Acta* **718**, 1–10 (2012).

33. E. G. Bligh, W. J. Dyer, A rapid method of total lipid extraction and purification. *Can. J. Biochem. Physiol.* **37**, 911–917 (1959).

34. J. Erde, R. R. O. Loo, J. A. Loo, Enhanced FASP (eFASP) to increase proteome coverage and sample recovery for quantitative proteomic experiments. *J. Proteome Res.* **13**, 1885–1895 (2014).

35. F. Pozzi, J. Arslanoglu, F. Galluzzi, C. Tokarski, R. Snyder, Mixing, dipping, and fixing: The experimental drawing techniques of Thomas Gainsborough. *Herit. Sci.* **8**, 85 (2020).

36. F. Carò, S. A. Centeno, D. Mahon, Painting with recycled materials: On the morphology of calcite pseudomorphs as evidence of the use of wood ash residues in Baroque paintings. *Herit. Sci.* **6**, 3 (2018).

37. N. Garnier, C. Rolando, J. M. Høtje, C. Tokarski, Analysis of archaeological triacylglycerols by high resolution nanoESI, FT-ICR MS and IRMPD MS/MS: Application to 5th century BC–4th century AD oil lamps from Olbia (Ukraine). *Int. J. Mass Spectrom.* **284**, 47–56 (2009).
